# Signal Pathways and microRNAs in Osteosarcoma Growth and the Dual Role of Mesenchymal Stem Cells in Oncogenesis

**DOI:** 10.3390/ijms24108993

**Published:** 2023-05-19

**Authors:** Natalia Todosenko, Igor Khlusov, Kristina Yurova, Olga Khaziakhmatova, Larisa Litvinova

**Affiliations:** 1 Center for Immunology and Cellular Biotechnology, Immanuel Kant Baltic Federal University, 236001 Kaliningrad, Russia; tod_89@mail.ru (N.T.); khlusov63@mail.ru (I.K.); kristina_kofanova@mail.ru (K.Y.); olga_khaziakhmatova@mail.ru (O.K.); 2 Laboratory of Cellular and Microfluidic Technologies, Siberian State Medical University, 2, Moskovskii Trakt, 634050 Tomsk, Russia

**Keywords:** osteosarcoma, mesenchymal stem cells, microRNAs, treatment

## Abstract

The major challenges in Osteosarcoma (OS) therapy are its heterogeneity and drug resistance. The development of new therapeutic approaches to overcome the major growth mechanisms of OS is urgently needed. The search for specific molecular targets and promising innovative approaches in OS therapy, including drug delivery methods, is an urgent problem. Modern regenerative medicine focuses on harnessing the potential of mesenchymal stem cells (MSCs) because they have low immunogenicity. MSCs are important cells that have received considerable attention in cancer research. Currently, new cell-based methods for using MSCs in medicine are being actively investigated and tested, especially as carriers for chemotherapeutics, nanoparticles, and photosensitizers. However, despite the inexhaustible regenerative potential and known anticancer properties of MSCs, they may trigger the development and progression of bone tumors. A better understanding of the complex cellular and molecular mechanisms of OS pathogenesis is essential to identify novel molecular effectors involved in oncogenesis. The current review focuses on signaling pathways and miRNAs involved in the development of OS and describes the role of MSCs in oncogenesis and their potential for antitumor cell-based therapy.

## 1. Introduction

Osteosarcoma (OS) is a genetically heterogeneous primary malignancy of long bones in children/adolescents (first peak at age 18 years); it may develop secondarily in patients with Paget’s disease and after radiotherapy (second peak at age 60 years) [1]. OS is the third most common primary bone tumor after chondrosarcoma and chordoma. The overall incidence is 3.4 cases per million per year worldwide [2].

OS is characterized by impaired immune response, pathological structure of bone tissue, and development of metastases, mainly in lung tissue (in 80% of cases). Depending on the characteristics of the tumor and the characteristics of immature osteoid tissue formed by OS cells, as well as the predominant direction of stromal differentiation of tumor cells, osteoblastic, fibroblastic, chondroblastic, small cell, telangiectatic, highly differentiated superficial and extraskeletal OS can be distinguished [3]. Depending on the histologic picture, OS distinguishes between high-grade, intermediate-grade, and low-grade malignancy [4]. The wide heterogeneity of OS is associated with point mutations and deletions in more than 80 genes [5]. Normally, OS is a term that refers to a high-grade intramedullary tumor that accounts for 85% of all cases in childhood and adolescence [6].

The earliest occurrence of OS dates back to the Triassic period (Amnio reptile, 240 million years old, Pappochelys rosinae) [7]. Nevertheless, OS is currently one of the most complex diseases in the field of oncologic orthopedics and is the focus of modern medical research. However, therapeutic options for bone sarcomas have not changed significantly since the late 1970s [8]. Surgical resection, radiation therapy, and chemotherapy (doxorubicin, ifosfamide, methotrexate, cisplatin) are common treatments for sarcomas [9]. However, chemotherapeutic approaches to the treatment of OS are effective only for localized OS. Progressive, metastatic, and recurrent OS are associated with the development of chemoresistance and poor prognosis [10]. More than 30% of patients with localized OS and more than 80% of patients with metastatic/recurrent disease die from the formation of resistant tumor subclones [11].

Since the introduction of multimodality chemotherapy into the treatment regimen for high-grade OS, the probability of disease-free survival has increased from 10–20% with surgery to 60% [2] with combination therapy. Radical treatment combines surgery with multimodality (combined) preoperative and postoperative chemotherapy with cytostatics (cisplatin, doxorubicin, high-dose methotrexate/ifosfamide, ifosfamide/etoposide, regorafenib, gemcitabine/docetaxel, carbozantinib, pazopanib), which aims to reduce single-drug toxicity and suppress drug resistance [12].

Nevertheless, the search for specific molecular targets and promising innovative approaches in OS therapy, including drug delivery methods, is an urgent problem [10]. Modern regenerative medicine focuses on harnessing the potential of mesenchymal stem cells (MSCs) because they have low immunogenicity [13]. This is mainly due to the fact that MSCs possess molecules of the major histocompatibility complex [14] to a small extent and have an immunosuppressive effect [15].

The use of cultured human bone marrow-derived MSCs (BM-MSCs) in conjunction with a bone marrow graft after bone tumor resection does not increase the risk of local recurrence [16].

However, targeted stem cell therapy for OS is not currently available. This is associated with a known risk of OS recurrence, tumor spread, and metastasis with MSC [17,18]. At the same time, there are reports of several preclinical studies that may improve the prognosis of disease progression in patients [19,20]. In addition, MSCs can be modified for delivery [21] and/or expression of tumor drugs and are used as mesenchymal killers [16].

Previously, we discussed the issues of therapeutic modulation of MSC to correct osteoporosis [22].

A better understanding of the complex cellular and molecular mechanisms of OS pathogenesis is essential to identify novel molecular effectors involved in oncogenesis and possible MSCs transformation into OS cells. Indeed, MSCs can transform into OS cells [23]. Therefore, knowledge of the signaling pathways and molecules that regulate the fate of OS cells may be important to understand the original molecular mechanisms of tumorigenesis in healthy stem cells.

The current review focuses on signaling pathways and miRNAs involved in the development of OS and describes the dual role of MSCs in sarcomogenesis and their potential for antitumor cell-based therapy.

## 2. Molecular Basis of OS Pathogenesis

### 2.1. The Role of miRNAs in the Pathogenesis of OS

MicroRNAs (miRNA, miR) are small, single-stranded, non-coding, highly conserved RNA molecules that regulate gene expression at the post-transcriptional level. miRNAs regulate up to 60% of genes encoding human proteins and modulate the levels of proteins involved in cell development, proliferation, differentiation, and apoptosis [24].

miRNAs are transcribed as monocistronic or polycistronic stem-loop RNA structures. Polycistronic transcripts contain clusters of multiple collinear immature miRNAs. After several processing steps, each individual miRNA transcript gives rise to a miRNA duplex of 20–22 nucleotides that forms a hairpin. One of the hairpin chains is involved in gene regulation, while the other, the “passenger thread”, is less active and is degraded. Active miRNA is preferentially incorporated into the RNA-induced silencing complex (RISC), which recognizes specific mRNA targets by complementary binding to the 3’-untranslated region (3’-UTR), mainly via the “seed” sequence, destabilizes or blocks mRNA translation (post-transcriptional gene silencing), and reduces their expression [24].

miRNAs with identical start sequences can target the same mRNA and are grouped into miRNA families. On the other hand, the same miRNA can target multiple mRNAs [25].

Many studies have shown that miRNAs are abnormally regulated in human malignancies, including OS, and act as a novel class of oncogenes or tumor suppressors. Literature data on the role of miRNAs in various OS cell lines suggest an important role of miRNAs in the progression of OS [26,27]. Other studies suggest that miRNAs play a central role in the pathogenesis of OS [24].

In this review, we focus on the major signaling pathways of OS cells and regulatory miRNAs involved in their molecular control.

### 2.2. Signaling Pathways and Regulatory miRNAs in OS

The pathogenesis of OS may be related to an alteration in the way MSCs differentiate into mature osteoblasts [28]. Abnormal expression of oncogenes and tumor suppressor genes caused by genetic and epigenetic events disrupts the regulation of key cellular signaling pathways, contributing to the conditions for the development of OS [29,30].

Unique features of the miRNA expression profile have been investigated in OS metastases as diagnostic biomarkers. The potential mechanisms by which miRNAs are involved in OS metastasis have also been the subject of much research [25].

At OS, dysregulation of many signaling pathways occurs in the bone microenvironment, including fibroblast growth factor (FGF), transforming growth factor β (TGF-β), insulin-like growth factor 1 (IGF1), bone morphogenetic proteins (BMPs), VEGF, hypoxia-induced factor (HIF1), a family of Wingless-type (WNT), Hedgehog (Hh), NOTCH MMTV integration sites involved in modulating self-renewal, differentiation, growth, drug resistance, and/or metastatic activity of OS and cancer stem cells (CSCs) [31,32].

Hypoxia-induced HIF1α activates the SDF1-CXCR4 signaling axis [33], and SDF1 ligand induces chemotaxis through membranes, adhesion to endothelial cells, and expression of metalloproteinases 2 and 9 (MMP-2, MMP-9), which are associated with metastasis OS [34]. CXCR4 expression correlates with VEGF expression and poor OS survival [35]. CXCR4-positive malignant cells have been found to be highly likely to metastasize to organs that express SDF1 (CXCL12) (bone marrow). The unique microenvironment of the bone marrow attracts and keeps tumor cells of diverse origins alive, and local BM-MSC interaction/recruitment promotes primary OS growth/invasion [36].

The master regulator of epithelial-mesenchymal transition (EMT) and tumor progression is TGF-β (and the downstream transcription factors Snails, ZEBs, Twist), whose levels correlate with grade (stage), chemoresistance, and the presence of metastases in OS [37,38]. TGF-β has been shown to regulate VEGF and connective tissue growth factor (CTGF) [39]. VEGF is associated with reduced disease-free survival in patients with OS [40]. CTGF promotes angiogenesis, increases MMP-2/3 expression and cell migration in OS [41,42], increases drug resistance [43], and regulates VEGF production by fibroblasts in OS [44]. TGF-β production by OS cells controls recruitment and differentiation of infiltrating immune cells and creates a local immune-tolerant microenvironment that promotes tumor progression [45].

PDGF (platelet-driven growth factor) maintains cancer stem cell phenotype (self-renewal, invasion, resistance to chemotherapy) [46] in OS [47].

The PI3K/Akt pathway is widely recognized as one of the major oncogenic factors in OS [48,49]. The Wnt/β-catenin pathway is activated at OS and plays a key role in oncogenesis [50,51], metastasis [52], angiogenesis, and immunological surveillance [53,54]. Abnormal activation of the Hedgehog (Hh) pathway in OS has been confirmed [55] and strategies targeting this pathway are being actively developed [56,57]. Promising results have also been obtained in studying the prognostic impact of p16 [58], HER2 [59,60], and β-catenin [61,62] expression using OS.

Gene set variation analysis (GSVA) and gene set enrichment analysis (GSEA) showed that the EMT pathway is strongly associated with the progression of OS, including the genes: LAMA3, LGALS1, SGCG, VEGFA, WNT5A, MATN3, ANPEP, FUCA1, and FLNA [63]. In addition, the formation of the tumor immunological microenvironment in OS is regulated by lipid metabolism-associated genes (LMRG), particularly ME1, ALOX15B, and GPD1 [64].

#### 2.2.1. Wnt/β-Catenin

Wnt ligands are a family of 19 secreted glycoproteins [65], some of which are capable of binding to one of ten transmembrane receptors of the Frizzled family (FZD1-10) or to single transmembrane receptors (LRP5, LRP6, ROR1, ROR2) to initiate canonical and non-canonical cascades [66].

The WNT/β-catenin signaling pathway is referred to as canonical WNT. In the absence of ligands, the WNT/β-catenin pathway is inactive and β-catenin is sequestered by the protein complex for degradation. This complex consists of scaffold proteins: Dvl, Axin1/2, WTX, and two kinases, CK1α and GSK3β, that sequentially phosphorylate β-catenin and promote its ubiquintylation and subsequent proteasomal degradation [65,67,68].

Canonical WNT signaling via Frizzled and LRP5/6 receptors promotes β-catenin-dependent transcription of TCF/LEF target genes (WNT/β-catenin signaling) and β-catenin-independent derepression of FOXM1, NRF2 (NFE2L2), YAP, and other proteins [WNT signaling/protein stabilization (STOP)].

Noncanonical WNT signaling via frizzled or ROR receptors activates dissheveled-dependent Rho-ROCK and Rac-JNK cascades [WNT/planar cell polarity (PCP) signaling]; G protein-dependent calcineurin-NFAT, CAMK2-NLK, and PKC cascades [WNT/G protein-coupled receptor (GPCR) signaling]; and receptor tyrosine kinase (RTK)-dependent PI3K-AKT and YAP/TAZ cascades (WNT/RTK signaling) [66].

Free YAP/TAZ is involved in the Hippo signaling pathway, which, when activated, induces hyperphosphorylation of YAP/TAZ and prevents nuclear translocation [69,70].

Secreted extracellular inhibitors of WNT ligands and activators of the canonical pathway include Dickkopf (DKK), secreted Fzd-related protein (SFRP), Wnt inhibitory factor 1 (WIF1), sclerostin (SOST), insulin-like growth factor binding protein 4 (IGFBP4) [65], transmembrane molecules ZNRF3 and RNF43, 7-transmembrane receptors LGR4, LGR5, LGR6 [68], and gene naked cuticle homolog 2 (NKD2) [71].

WNT signaling regulates target cell self-renewal, metabolism, survival, proliferation, and epithelial-mesenchymal transition (EMT), and also interacts with FGF, Hedgehog, Notch, and transforming growth factor β (TGF-β). WNT/β-catenin signaling controls cellular signaling cascades, including epidermal growth factor receptor (EGFR), Hippo/YAP, nuclear factor kappa-B (NF-kB), Notch, Sonic Hedgehog, and the PI3K/Akt pathway, which are involved in cancer development [72,73,74].

Because intracellular and intercellular WNT signaling networks control embryogenesis and homeostasis, genetic alterations in WNT signaling molecules have been implicated in the pathogenesis of several human cancers [66].

The WNT pathway has been shown to play an important role in the development of OS, but due to the complexity of this pathway, the current data are not fully understood. Specifically, WNT/β-catenin OS promotes cell invasion and migration, and WNT has a direct impact on OS resistance to chemotherapy [75]. Recent studies highlight the role of the WNT/β-catenin pathway in angiogenesis and immunological surveillance, processes involved in the metastatic spread of OS [50].

There is high expression of β-catenin in OS tissues, which is associated with poor prognosis and lung metastasis [76,77].

Another study highlights that inactivation of the WNT/β-catenin pathway plays a key role in the development of OS [78].

In addition, frequent deletions of genes related to the WNT pathway have been described in patients with OS [79].

Activation of the canonical WNT/β-catenin pathway is associated with suppression of apoptosis in MG63 cells [80]. Cell motility (associated with OS progression and metastasis) and cell apoptosis are regulated in part by the Rho/ROCK signaling pathway [80].

Cancer stem cells (CSCs) are characterized by persistent activation of highly conserved signaling pathways, including Notch, Hedgehog, and Wnt [81]. Constitutive activation of WNT/β-catenin signaling in OS CSCs compared with native OS cells has been described [82]. Liu et al. highlighted the upregulation of the properties of CSCs in OS by β-catenin in vitro [77].

Canonical transmission

- NKD2

Microarray profiling revealed that the Cuticle Naked Homologue 2 (NKD2) gene is persistently downregulated in metastatic and recurrent OS tumors, whereas overexpression is associated with less aggressive disease. NKD2 is a negative regulator of Wnt signaling and is located downstream of LRP5 [71].

miRNAs: miR-130b is overexpressed in human OS and activates proliferation and inhibits apoptosis in OS cells by binding NKD2 and subsequently suppressing Wnt signaling [83]. miR-346 targets GSK-3β to activate β-catenin [84].

- TCF/LEF

TCF-1, which is the major binding complex of β-catenin and partially prevents apoptosis of OS cells, has been shown to be overexpressed in OS. At the same time, p53 plays a critical role in the expression of TCF-1 [85]. TCF-7 is also highly expressed in OS cells [86].

miRNAs: Forced expression of miR-192 inhibited proliferation, invasion, and migration of U2OS and MG63 cells and induced cell apoptosis [86]. According to the results of Western blotting and luciferase analysis, TCF-7 was identified as a target of miR-192. According to the bioinformatics analysis, TCF-4 is a direct target of miR-4695-5p (low expression in OS) in OS cells and acts as a tumor suppressor [87].

- FOXM1

FOXM1 (foxhead box M1) is highly expressed in OS cells [88] and is associated with cancer cell proliferation, migration, and invasion. Activation of FoxM1 transcription is thought to increase oncogenicity and promote tumor cell proliferation [89] and maintain several carcinogenic signaling pathways, including Wnt, SMAD3, and NF-kB, through interaction with other proteins [90].

miRNAs: It has been shown that miR-320a and miR-320b function as OS inhibitors [91,92]. Overexpression of these miRs promotes downregulation of FOXM1 expression in OS cells [93]. Low expression of miR-361-5p was observed in OS tissues and cell lines. The target for miR-361-5p is FoxM1 [94]. At the same time, similar low expression of miR-361 was observed in OS cell lines (MG63, Saos-2), and its target genes were VEGF, FoxM1, and Twist, which are associated with cell cycle, apoptosis, cell growth, and metastasis [95].

In OS cell lines (U2OS, MG63, Saos-2), miR-216b expression was low. miR-216b has an antitumor role by targeting FoxM1 in OS [96] and suppressing the migratory and invasive potential of cells. In addition, miR-216b expression synergistically inhibits cytotoxicity and stimulates apoptosis in OS cells by increasing Bax expression and decreasing Bcl-2 expression [96]. Low expression of miR-134 was observed in OS cell lines (U2OS, MG63). At the same time, bioinformatics analysis revealed that the 3’-UTR region of the FoxM1 gene contains binding sites for miR-134 [97]. Moreover, FoxM1 is a target gene for miR-197 in OS cells [98]. Moreover, upregulation of miR-370 inhibited cell growth and OS metastasis by downregulating FoxM1 expression [99].

Interestingly, FoxM1 is associated with cell cycle regulatory genes with maximal expression in the S phase and G2/M phase (E2F, E2F1, E2F4, E2F6, GABPA) in the OS (U2OS) cell line [100].

- E2F1

One of the cell cycle regulators is the transcription factor E2F1 (E2F Transcription Factor 1).

miRNAs: Duplicate analysis of luciferase confirmed the existence of a regulatory relationship between miR-185-3p and E2F1. Overexpression of miR-211-5p was found to decrease the expression of E2F1 in OS cells [101]. E2F1 is a target for miR-329 and regulates epithelial-mesenchymal transition (EMT), which has been confirmed in tissue samples from patients with OS [102]. Moreover, miR-320 can directly regulate E2F1 expression in U2OS cells [103].

- E2F5

E2F5 (E2F transcription factor 5) is a family of E2F proteins. E2F5 can directly bind to cell cycle-associated genes and promote cell cycle progression in human cancer [104].

miRNAs: It has been reported that E2F5 is disrupted in OS by direct binding of miR-154-5p in OS cells [105]. Overexpression of miR-154-5p significantly reduced the expression of E2F5 in MG63 cells, resulting in cell cycle arrest in the G0/G1 phase [105].

The transcription factor E2F5 is the downstream miR-513b-5p gene. Overexpression of miR-513b-5p suppressed proliferation, migration, and invasion of OS cells [106].

- E2F3

The E2F family is an important regulator of the cell cycle. The transcription factor E2F3 (E2F transcription factor 3) is oncogenic in cancer [107].

miRNAs: A high expression level of has_circ_0008934 was found in OS cell lines (Saos-2, MG63). Suppression of has_circ_0008934 increased apoptosis, blocked cell cycle progression, and impaired the ability of Saos-2 cells to migrate, invade, and proliferate. Has_circ_0008934 directly binds to miR-145-5p and suppresses E2F3 expression, promoting tumorigenesis of MG63 cells in nude mice [108].

It was found that miR-16-5p interacts with the 3’-UTR of E2F3 and represses the amount of E2F3 mRNA. According to the report analysis, E2F3 is the target of miR-16-5p [109]. In addition, E2F3 acts as a target for miR-124-3p [110], miR-874 [111], miR-152, and miR-145-5p [112] in OS tissue.

- RB1-E2F

Retinoblastoma tumor suppressor protein 1 (RB1) [113] forms a suppression complex with E2F family transcription factors and negatively regulates G1/S transition during the cell cycle by inhibiting E2F activity, E2F1, E2F2, and E2F3a (link to RB) [114]. Low expression of RB1 was detected in the MG63 cell line [115].

miRNAs: According to TargetScan and luciferase report analysis of the 3’-untranslated region, miR-17 is directly controlled by the RB1 gene and affects BMP9-induced osteoblast differentiation [116]. High expression of miR-17 was detected in the cell line OS (MG63) [117].

- NRF2 (NFE2L2)

NRF2 (Nuclear Factor Erythroid 2-Related Factor 2) is considered a key factor in cell protection and survival. High expression of NRF2 was observed in biopsy samples from patients with OS and was associated with poor clinical outcome and disease-free survival [118]. NRF2 levels increased after α-irradiation of human OS cells. At the same time, high NRF2 expression in cell lines 143B and MG63 was associated with high expression of ABC-transporter and high chemoresistance of cells [119].

miRNAs: miR-340-5p is able to significantly suppress the invasion and migration of OS cells by binding to NRF2 and mediating regulation of the PI3K/AKT pathway and epithelial-mesenchymal transition (OS) [120].

- YAP

Patients with OS have been found to have high expression of the protein YAP in tumor tissue [121], which is associated with poor prognosis [122].

According to functional analysis, nuclear activity of YAP correlates with actin cytoskeleton stability, which is associated with the ability of cells to migrate and invade, and specifically induces a migratory phenotype of OS cells [123].

miRNAs: It was found that miR-515-5p is able to inhibit stemness of OS CSCs by targeting the level of YAP mRNA expression [124]. miR-129-5p interacts with YAP1 and inhibits cell proliferation, migration, and invasion during OS development (in U2OS, MG63 cell lines) [125]. Similarly, miR-375 targets YAP1 in OS cells [126]. miR-195-5p was shown to bind to the 3’-UTR of the YAP gene in OS cells [127]. In addition, luciferase double report analysis showed high binding efficiency between miR-1285-3p and YAP1 in OS cells [128]. YAP1 was also identified as an inhibitory target of miR-132-3p in OS cells (MG63, 143B, Saos-2, HOS) [129]. Bioinformatics and luciferase report assays have shown that miR-625 targets YAP1 in OS [130].

Noncanonical transmission

- Rho-ROCK

Rho-associated kinase (ROCK) is an important downstream effector of the small GTPase Rho that acts as a molecular switch by binding GTP (active) and GDP (inactive) to regulate cell survival, proliferation, and cytoskeletal organization, and to induce changes in cell shape, morphology, adhesion, and movement. Two isoforms are known: ROCK1 and ROCK2. High ROCK expression is associated with cancer progression, metastasis, and poor prognosis [131]. Inhibition of ROCK leads to cell death, impairs angiogenesis, and reduces metastasis in oncology, including OS [132].

ROCK1 is an important modulator of cell polarity, cell morphology, regulation of gene expression, cell proliferation, differentiation, apoptosis, stem cell formation, and tumorigenesis [133]. ROCK1 has been shown to be activated in OS tissues [134]. Like ROCK1, ROCK2 is also activated in OS and is a critical factor in OS cell migration. It was found that there is a functional relationship between ROCK2 and YAP that regulates OS migration/metastasis [135].

miRNAs: Low expression of miR-144 was found to be associated with proliferation, apoptosis, invasion, and metastasis of the OS F5M2 cell line [136]. Overexpression of miR-144 leads to inhibition of tumor cell growth and invasion in vitro, and contributes to suppression of OS metastasis in vivo [131,137]. The putative targets for miR-144 are ROCK1 and ROCK2.

Another miR that is downregulated in OS cells is miR-340. Ectopic expression of miR-340 suppressed ROCK1 by directly binding to its 3’-untranslated region, resulting in significant inhibition of cell proliferation, migration, and invasion in OS cell lines in vitro, and OS tumor growth in a mouse xenograft model [134]. Similar results were obtained in the analysis of primary OS in children [138].

In addition, low miR-148a expression was associated with OS progression and poor prognosis. In Saos-2 and U2OS cell lines, the target of miR-148a was also found to be ROCK1. Studies have shown that miR-miR-148a acts as a tumor suppressor at OS by targeting ROCK1 and exerting a suppressive effect on the proliferative, invasive, and migratory capabilities of Saos-2 and U2OS cells [139].

Reduced expression of miR-139 in OS has been identified as a tumor suppressor of tumorigenesis, and its suppressive effect on tumor cell proliferation and invasion has been demonstrated by targeting ROCK1 [140].

Studies have identified a number of miRs that exhibit antitumor activity due to a direct effect on ROCK1 OS: miR-335 [141,142], miR-145 [143], miR-129-5p [144], miR-214-5p [145], miR-101 [146], and miR-202-5p [133,147].

Receptor tyrosine kinase (RTK)-dependent PI3K-AKT cascade c-Met

MET belongs to a family of receptor tyrosine kinase oncogenes [148] that are overexpressed in OS (23.3%). An uncontrolled cluster analysis of miRNA expression profiles (based on the Sarcoma miRs Expression Database-S-MED) showed that OS forms a single cluster distinct from other sarcomas, e.g., synovial sarcomas, fibrosarcomas, gastroenteric stromal tumors, and malignant fibrous histiocytomas [149].

c-Met is a member of the RTK family (generally expressed in epithelial cells) and is an oncogene that activates multiple signaling pathways [150]. c-Met is considered a prognostic marker in cancer patients [151]. c-Met also (positively) regulates the progression of OS [152] through high expression in tumor cells [153].

miRNAs: miR-449b-5p suppresses the proliferative, migratory, and invasive capabilities of OS cells by directly targeting c-Met [154].

miR-454, which targets c-Met and can inhibit metastatic spread of OS cells, exhibits similar properties [152].

Low expression of miR-34a correlated with the metastatic potential of OS cells (SOSP-9607). It was found that miR-34a is able to specifically suppress the expression of c-Met gene in OS cells [155].

Under-expression of miR-876-5p positively correlates with late clinical stage and short overall survival in patients with OS. Suppression of miR-876-5p promoted proliferation, migration, and invasion in U2OS and MG63 cell lines. Overexpression of miR-876-5p inhibited the growth of OS tumors in in vivo mouse models [156].

Overexpression of miR-191a-3p in OS was associated with a significant reduction in cell growth in G1 arrest. Moreover, miR-191a-3p suppressed the expression of oncogenic and anti-apoptotic proteins, MET, mTOR, STAT3, MCL-1, and BCL-X, indicating an important regulatory role of miR-191a-3p in OS cell proliferation [149].

Other participants in the Wnt signaling pathway

- AXIN1

AXIN1 has a strong influence on Wnt/β-catenin signaling and is the central scaffolding protein responsible for the formation of the β-catenin destruction complex [157].

miRNAs: miR-31-5p targeting AXIN1 is expressed at low levels in OS tissues, leading to activation of the Wnt/β-catenin pathway and increased translocation of β-catenin to the nucleus, promoting proliferation, invasion, and tumorigenicity of OS cells [158].

- SOX4

Transcription factors, including the Highly Mobile Group (HMG) Box (SOX) proteins associated with sex-determining region Y (SRY), are involved in the regulation of certain biological processes [159].

SOX4 belongs to the transcription factor family SOX (SRY-related high-mobility group (HMG)-box) and regulates cell proliferation, migration, invasion, apoptosis, and epithelial-mesenchymal transition in cancer. Overexpression of SOX4 is associated with activation of PI3K, Wnt, and TGF-β signaling pathways [160].

miRNAs: Activated miR-25 [161], miR-188 [162] and miR-214 [163] inhibit OS cell proliferation, migration, and invasion by directly targeting SOX4. miR-132 inhibits cell growth and metastasis in miR cell lines by targeting SOX4 [164]. Ectopic expression of miR-363-3p [165] and miR-212 [166] suppressed proliferation, migration, and invasion of OS (U2OS, MG63) cell lines by targeting SOX4.

- STAT3

The expression of STAT3, β-catenin, c-Myc, TCF4, CyclinD1, and ROCK1 proteins is significantly increased in U2OS cell line [167].

miRNAs: miR-340-5p directly interacts with the 3’-untranslated region (3’-UTR) of the STAT3 gene and negatively regulates its expression. An increase in miR-340-5p expression can markedly inhibit the proliferation of U2OS cells in vitro and induce apoptosis [167]. High expression of miR-30d-5p in OS cells (U2OS, MG63) is associated with low levels of SOCS3 suppressor protein and activation of the JAT/STAT3 signaling cascade, and progression of the tumor process [168]. In addition, SOCS3 is also a target for miR-221-3p. At the same time, high levels of miR-221-3p in OS cells are associated with JAT/STAT3 activation, migration, invasion, and high viability of cancer cells [169].

- Dkk

The Dickkopf (Dkk) family is capable of inhibiting the Wnt/β-catenin signaling pathway [170]. Dkk-1 is thought to act as an inhibitory Wnt factor by directly binding to LRP6, causing abnormal bone metabolism. High expression of Dkk-1 has been observed in OS [171].

miRNAs: OS tissues show low expression of miR-107, which acts as a tumor suppressor and targets Dkk-1 [171].

- IGF2BP1

IGF2BP1 (Insulin-Like Growth Factor 2 Binding Protein 1) belongs to the highly conserved IGF2BP protein family and is highly expressed between zygote and embryonic stages. Moreover, abnormally high expression of IGF2BP1 is observed in various cancers, suggesting its oncogenic role [172]. High IGF2BP1 immunoreactivity has been associated with a high degree of OS tumor progression, the presence of metastases, relapse, and poor response to chemotherapy [172].

miRNAs: The downstream target for miR-150 in OS is IGF2BP1 [172].

- MYC

MYC is an important transcription factor for the Spral loop helix leucine zipper and functions as an important regulator of several cellular processes. In addition, MYC is recognized as an oncogene that is overexpressed in cancer, including OS [173].

miRNAs: Overexpression of miR-193b has been shown to contribute to the decrease in expression of MYC. It is suggested that miR-193b has an indirect effect on MYC expression by targeting other MYC regulatory proteins or miRNAs in OS [174].

- CCNE2

The cell cycle is regulated by a family of cyclin-dependent kinases (CDKs) linked to respective regulatory subunits of cyclin. Fluctuations in cyclin levels determine fluctuations in CDK activity that control cell cycle phase transitions [175]. The oncogenic activity of the cyclinE/CDK2 complex is observed in cancer. Cyclin E2 is encoded by the CCNE2 gene [176].

miRNAs: CCNE2, involved in G0/G1 cell cycle regulation, was downregulated by miR-34c in OS cells (transcriptome analysis). Targeting the oncogenic complex cyclinE/CDK2 has been proposed as a promising cancer therapy [177,178].

#### 2.2.2. Hippo/YAP/TAZ

The Hippo cascade is activated by various signals and plays a critical role in regulating cellular plasticity and tumor cell processes: proliferation, apoptosis, and migration [70]. Upon activation of the Hippo pathway, activated MST1/2 (mammalian St20-like kinases ½) phosphorylate and activate LATS1/2 factors (large tumor suppressors ½), leading to inhibition of YAP/TAZ (Yes-Associated Protein/paralog transcription coactivator with PDZ binding motif). Phosphorylation of YAP on Ser127 allows binding to the 14-3-3 protein, leading to its degradation via the ubiquinin proteasome pathway [179]. When the Hippo pathway is not involved, YAP/TAZ are not phosphorylated and can be translocated to the nucleus, where they act as co-transcription factors. The major DNA-binding partners of YAP are TEAD1-4 proteins (TEA-domain of DNA-binding transcription factor 1-4), which regulate cell proliferation, differentiation, and apoptosis [180]. Thus, the Hippo pathway plays an inhibitory role in regulating oncogenesis [181].

The molecular mechanism of overexpression of YAP in OS is thought to be related to the stem cell transcription factor SOX2 [182]. SOX2 maintains cancer cell stem cell function by regulating the Hippo pathway [183]. The YAP/TEAD axis has been reported to be involved in controlling the growth of OS primary tumors [121]. Overexpression of the mutant YAP protein in OS cells stimulates the development of lung metastases in vivo [123].

miRNAs: Studies have identified a pro-oncogenic effect of high expression of miR-624-5p in OS cell lines and tissue samples that aims to indirectly inhibit Hippo signaling activity (increase in phosphorylated YAP) through direct suppression of PTPRB (protein tyrosine phosphatase type B receptor) [184]. miR-224 is a phenocopy of TAZ and is activated upon TAZ overexpression, inhibiting the tumor suppressor SMAD4 (a key factor in the TGFβ pathway), and maintaining the proliferation and migration potential of OS cells [185].

Moreover, miR-135b induced by TAZ also stimulates metastasis in OS [186].

#### 2.2.3. TGF-β

Activation of the TGF-β pathway is associated with a protumor effect that stimulates the migratory and invasive activity of OS cells and leads to the appearance of lung metastases [187].

TGF-β is involved in regulating the biological function of malignant tumors in several ways, including Jagged1/Notch, Wnt, JAK2/Stat3, and PI3K/AKT/mTOR [188].

TGF-β is highly expressed in OS tissues (human) and OS cell lines [188]. TGF-β plays a role in modulating apoptosis of OS cells [189]. Suppression of TGF-β leads to cell cycle arrest in the S phase and increases the expression of markers associated with apoptosis. TGF-β accelerates EMT in OS cells and promotes the development of chemoresistance and lung metastases [190].

miRNAs: Overexpression of miR-181c has been shown to markedly suppress the proliferation, invasion, and migratory ability of OS cells (in vivo BALB/c mouse models) by modulating TGF-β signaling (suppression), specifically by regulating the transcription factor SMAD7 (negative regulator TGF-β) [191]. Suppression of SMAD7 promotes BMP-induced chondrogenesis and osteoblast differentiation from BM-MSC [192]. The study showed that SMAD7 is the target gene for miR-181c in OS cells [191].

High expression of miR-330-5p was found in OS cells, which was associated with distant metastasis and low overall survival. At the same time, miR-330-5p might contribute to the malignant progression of OS through the mutual modulation of SPRY2 and Tgfβ1/Smad signaling pathways [193].

miR-140-3p targeting TRAF6 was found to inhibit TGF-β-induced epithelial-mesenchymal transition, migration, and invasion of human OS [194].

It is hypothesized that miR-21 targets the TGF-β1 signaling pathway to promote cell proliferation of OS. Suppression of miR-RNA-21 inhibits OS proliferation and promotes PTEN and TGF-β1 protein expression in human osteoblast cells and human U2OS cell lines [195]. Moreover, treatment with a TGF-β1 inhibitor counteracts the inhibitory effects of miR-21 knockdown on cell proliferation of OS [196]. miR-21 in serum and bone tissue has been proposed as a marker for early diagnosis of OS and evaluation of distant lung metastases [197].

TGF-β1 was confirmed as a putative target of miR-26a-5p in OS [198].

MSCS: Human MSCs derived from umbilical cord blood can release exosomes containing miR-181c-5p [199].

- SMAD3

Smad3 is an important participant in the transforming growth factor β (TGFβ) signaling pathway, which can transport TGFβ signaling from the cell membrane to the nucleus and regulate the expression of target genes during proliferation. Abnormal expression and function of Smad3 leads to disruption of cell proliferation, differentiation, migration, and apoptosis, and promotes cancer development, progression, and metastasis [200].

It was found that OS cells can release TGF-β, which is suppressed by an inhibitor of TGF-β receptor type I and knockdown of Smad3. In addition, high expression of TGF-β, Smad2/3, and p-Smad2/3 was found in biopsy samples from a patient with OS [201]. Smad3 expression was detected in several OS cell lines and in a human osteoblast cell line [202].

SMAD3 is highly expressed in OS cells. According to transwell analysis, overexpression of SMAD3 significantly promoted the migration and invasion of OS cells [203]

miRNAs: miR-671-5p was significantly suppressed in OS tissues and cells. Overexpression of miR-671-5p in OS cells is associated with decreased migratory ability and invasion. Reporter analysis with two luciferases confirmed the presence of a target binding site between miR-671-5p and the 3’UTR of SMAD3. miR-671-5p can significantly inhibit the invasion and migration of OS cells by negatively regulating SMAD3 [203].

Low expression of miR-16-5p was also observed in human OS cells, adjacent tissues and cell lines. At the same time, overexpression of miR-16-5p resulted in inhibition of proliferation, migration, and invasion of OS cells. Studies have shown that overexpression of miR-16-5p inhibits processes involved in metastasis (proliferation, migration, invasion) and chemoresistance (to cisplatin) of OS cells by targeting Smad3 [202].

A significant decrease in miR-422a expression is also observed in tissues and cell lines from OS compared to control. Overexpression of miR-422a is able to inhibit cell proliferation and invasiveness, as well as enhance cisplatin-mediated (and paclitaxel-mediated) apoptosis in OS cells. Since miR-422a targets TGF-β2, the mediated expression and activation of downstream Smad2/3 molecules in OS cells is also regulated by this miR [204].

#### 2.2.4. Notch

Notch signaling plays an important role in development and cell fate determination [205]. The binding of the ligand to the receptor leads to proteolytic cleavage of Notch by y-secretases, accompanied by the release of the intracellular 80 kDa Notch domain (NICD), followed by its translocation to the nucleus and activation of the transcription of downstream target genes [206].

Dysregulation of the Notch pathway has been shown to play a stimulatory/oncogenic role and promote osteosarcoma metastasis through p-Erk phosphorylation [207,208].

Inhibition of Notch activation was found to lead to apoptosis of OS cells resistant to chemotherapy [209].

The results of several studies using cell samples from patients with untreated OS showed high expression of the NOTCH2 and JAG1 genes (in most of the biopsies examined), and increased expression of the NOTCH1 and DLL1 genes (in one patient) [210]. This contrasts with the results of another study that found increased mRNA expression of the NOTCH1 and JAG1 (but not NOTCH2) genes in human OS cells [211]. In both studies, Notch target genes, particularly HEY1 and HEY2, were increased in OS samples, confirming activation of canonical Notch signaling. Notch signaling may also play a role in tumor invasiveness, as OS cell lines with the ability to metastasize have higher mRNA expression of NOTCH1, NOTCH2, and DLL1 genes, as well as the Notch target gene HES1 [212].

Moreover, conditional induction of Notch1 NICD in mature osteoblasts leads to spontaneous development of OS in mice aged 5 to 14 months [213]. Tumorigenesis and progression were accelerated by the loss of p53 and required activation of canonical Notch signaling.

High Notch1 expression has been shown to be associated with active autophagy in human OS cells. Moreover, overexpression of Notch1 can inhibit cell proliferation of OS and promote apoptosis and autophagy by regulating the PI3K/Akt/mTOR pathway (by inhibiting phosphorylation), which acts as a carcinogenic factor (trigger of carcinogenesis) [206].

Autophagy has been shown to play a dual role in regulating cell death. Mild (moderate) autophagy protects cells from harmful conditions, and rapid autophagy induces programmed cell death (autophagic cell death) [214].

Interestingly, OS cells have high expression of cell migration-inducing protein (CEMIP), which has been associated with poor prognosis in OS patients. Mechanistically, CEMIP promotes the growth and metastasis of OS cells by activating the Notch signaling pathway. Concurrently, suppression of CEMIP was associated with a decrease in protein expression and activation of the Notch/Jagged1/Hes1 pathway in vitro and in vivo [215].

Notch has been found to interact with HIF1α, leading to hypoxia-induced growth of tumor cells [216]. At the same time, the Notch signaling pathway is a link between angiogenesis and self-renewal of cancer cells [217].

It is possible that somatic activating mutations in Notch receptors, ligands, or target genes play a role in tumor development. As a result, components of the Notch pathway may become future therapeutic targets OS [218].

miRNAs: miR-34c is an effector of the tumor suppressor p53 and a negative regulator of the Notch pathway in osteoblast differentiation. At the same time, miR-34c inhibits proliferation and invasion of metastatic OS cells, resulting in prolonged overall survival in an orthotopic xenograft model. miR-34c regulates the transcription of Notch signaling cascade genes (NOTCH1, JAG1, HEY2). Thus, miR-34c can suppress the growth and progression of OS in vivo [219]. Moreover, low expression of miR-34c-5p, which regulates proliferation, migration, and invasion of OS cell lines, is detected in OS cells [220].

miRNA profiling in OS tissue samples revealed a 10-fold increase in miR-199b-5p expression, which was confirmed by PCR [221].

High miR-199b-5p expression in OS tissues and cells (MG63, U2OS) is associated with a high rate of distant metastasis and low overall survival in patients with OS [222]. miR-199b-5p plays an important role in Notch signaling in OS [221,223].

Recently, inhibition of Notch and HES1 signaling has been proposed as a potential therapeutic strategy to prevent metastasis in human OS [221].

Low expression of miR-1296-5p was detected in OS samples and was associated with large tumor size and distant metastases, which was reflected in the short survival time of patients. Overexpression of miR-1296-5p suppressed proliferation, migration, and invasion of OS cells through a direct effect on the Notch2 receptor (NOTCH2) [224].

#### 2.2.5. HIF1α

Hypoxia-induced factor 1α (HIF1α) is associated with the development and progression of malignancies, including OS [225,226]. High expression of HIF1α is an unfavorable factor for OS and serves as a predictor for OS [227].

miRNAs: miR-338-3p is able to downregulate the activity of the HIF-1/Rap1/PI3K-Akt pathway and reduce the proliferation potential of OS cells [228]. The target of miR-186 is HIF1 in OS cells [229].

#### 2.2.6. ErbB

The ErbB family of receptors, including EGFR, HER2, HER3, and HER4, are involved in activation of the PI3K/Akt and MAPK signaling pathways, and are potential targets for treatment in OS [230].

The ErbB receptor family is overexpressed in malignant neoplasms, including OS, and is associated with poor prognosis: distant metastases and no response to chemotherapy [231].

- EGFR (epidermal growth factor receptor)

High expression of EGFR is observed in tissues from OS in association with tumor progression [232]. EGF-induced EGFR phosphorylation in OS cell lines was found to activate MMP9 and promote invasion of OS cells [233].

miRNAs: miR-7 specifically interacts with EGFR and reduces the migration and invasion of OS cells [234]. The results of the dual luciferase report analysis showed the presence of a specific binding site for miR-3184-5p in the 3’UTR region of EGFR, confirming that EGFR is a direct target of miR-3184-5p in OS (143B, U2OS) cells [232]. miR-143 inhibits EGFR signaling through downstream ERK/MAPK signaling cascades by controlling MMP9 expression in OS [233]. In addition, EGFR is a target of miR-141-3p in OS [235]. miR-491-5p reduced EGFR expression in OS cells [236].

- HER2 (human epidermal growth factor receptor 2)

HER2 belongs to the ErbB receptor family [230]. Overexpression of HER2 is significantly associated with poor outcome in patients with OS and should be evaluated as a prognostic marker at diagnosis and after surgery [237]. In addition, positive expression of the HER2 gene is associated with OS metastasis [60] and histopathologic evidence of fibrous tissue, differentiation, and the presence of large areas of osteoid [238].

Tumor-associated expression of HER2 protein in the context of oncologic progression is the basis of a clinical trial (NCT04147819) [239]. Inhibition of HER2 by drugs (lapatinib) blocks proliferation of U2OS and MG63 cells and induces apoptosis [240].

miRNAs: Overexpression of miR-199b-5p is strongly associated with stage, distant metastasis, and poor prognosis OS. miR-199b-5p is associated with HER2-mediated OS progression [222].

#### 2.2.7. PI3K/Akt/mTOR

PI3KR1 is thought to play the role of a tumor suppressor. Activation of PI3K is triggered by the binding of p85 to an activated receptor tyrosine kinase (RTK). PI3K/Akt/mTOR are signaling pathways closely associated with apoptosis and cell proliferation and are usually highly upregulated OS [241,242,243]. The PI3K/Akt/mTOR pathway has emerged as one of the most common abnormalities in the metastatic behavior of OS, as activation of this signaling cascade accelerates cell metastasis and is associated with poor prognosis OS [244,245]. Suppression/deletion of PTEN activates the PI3K/AKT pathway, leading to cancer development, invasion, and metastasis [197].

The Ras/PI3K/Akt pathway has been shown to positively regulate oncogenic factor TGF-β levels in OS mice [246], suggesting that the TGF-β/PI3K/Akt axis is responsible for OS progression [188].

miRNAs: Expression of miR-21-5p has been found to be increased during osteogenic differentiation of MSCs [247]. miR-21-5p is considered an oncogene that promotes tumor proliferation and invasion [248]. MSCs-derived exosomes might be rich in miR-21-5p, which further affects the biological properties of OS. At the same time, exosomes protect miR-21-5p from degradation by RNase in the microenvironment associated with OS. The target gene for miR-21-5p is PI3KR1; moreover, miRNA is responsible for encoding an important regulatory subunit of PI3K, p85α [240]. PI3KR1 was found to be upregulated and the PI3K/Akt/mTOR pathway was inhibited when OS cells were treated with MSC-derived exosomes containing a miR-21-5p inhibitor [249].

miR-340-5p expression is significantly reduced in OS tissues and cells, which is associated with shorter overall survival and malignant clinical and pathological features of patients with OS. miR-340-5p targets NRF2 and negatively regulates PI3K/Akt and EMT signaling, which has a (preclinical) antitumor effect at OS [120].

It was found that miR-22 inhibits autophagy and prevents chemoresistance in MG63 cell line by suppressing the expression of PI3K, Akt, and mTOR at the mRNA level, and the expression of PI3K, phosphorylated (p)-Akt, and (p)-mTOR at the protein level [250].

- elF4E (eukaryotic transcription initiation factor 4E)

elF4E is a cap-binding protein that specifically recognizes the 5’ cap of m7G through stacking interactions with two tryptophan residues between two conserved tryptophans in the cap-binding pocket of elF4E. elF4E plays a key role in mRNA recruitment to control translation initiation and rate [251]. In addition, elF4E acts as an oncogene in vivo. Numerous studies have shown that elF4E/phosphorylated elF4E is present in various cancers, and overexpression of elF4E is observed in 30% of human cancers [252]. Activation of the mTOR pathway by phosphorylation of 4E-BP1 decreases the binding of elF4E to 4E-BP1, resulting in hyperactivation of elF4E [253,254] and also induces protein synthesis, ribosome biogenesis, cell proliferation and survival, metastasis, and angiogenesis through activation of ribosomal protein S6 kinase 1 (S6K1) [255].

miRNAs: In OS cells, decreased expression of miR-496 and increased expression of elF4E was observed. Expression of miR-496 correlated positively with survival of OS patients and had a suppressive effect on OS cell proliferation, migration, and invasion in vitro and tumor growth in vivo. elF4E may be a direct target of miR-496 [256].

- HMGB1 (High-Mobility Group Box 1)

HMGB1 is the gene encoding the non-histone protein HMGB1, which increases transcription and acts as an extracellular signaling molecule during tumor progression [257]. HMGB1 promotes oncogenesis: cell proliferation, angiogenesis, apoptosis exit, tissue invasion, and metastasis. HMGB1 protects cells from oxidative stress-induced apoptosis by promoting Beclin-1-mediated autophagy. High expression of HMGB1 mRNA and HMGB1 protein was observed in OS tissues (in cell line MG63) [258], including Saos2, SW1353, U2OS, and HOS [259]. HMGB1-induced autophagy leads to increased chemoresistance in OS [260,261].

miRNAs: Decreased expression of miR-505 was observed in OS tissues, which was associated with poor clinical prognosis in patients. miR-505 was found to inhibit proliferation, migration, and invasion of OS cells by regulating HMGB1 [262,263]. Low expression of miR-1284 was also detected in the U2OS and MG63 cell lines, whose target genome is also HMGB1. Overexpression of miR-1284 inhibited cell proliferation, cell migration, and altered protein expression of epithelial-mesenchymal transition (EMT)-related genes (E-cadherin, N-cadgenrin, vimentin), which was reversed by overexpression of HMGB1 [264]. HMGB1 plays an important role in facilitating autophagy and promotes drug resistance in OS cells. miR-22 paired with the 3’-UTR of HMGB1 suppressed the expression of this gene and blocked autophagy during chemotherapy in OS cells in vitro and also inhibited proliferation, migration, and invasion of OS cells [265]. However, low plasma levels of miR-22 were observed in patients with OS, correlating with large tumor size, late clinical stages, positive distant metastases, and poor tumor response to preoperative chemotherapy. Multivariate analysis showed that low miR-22 expression in plasma was an important independent predictor of poor prognosis OS [266]. Moreover, overexpression of miR-22 at the cellular level impairs proliferation and EMT (OS) by targeting the Twist1 signaling pathway [267]. In addition, a small amount of miR-372-3p, which interacts with the 3’-UTP of HMGB1, has been detected in OS cells [268]. There are also binding sites between miR-142-3p, miR-129-3p, miR-32-5p, and HMGB1 [259,269].

#### 2.2.8. RUNX2/CDK4

Abnormal expression of transcription factor 2 (RUNX2) is a key pathological feature of OS. Elevated RUNX2 levels can transcriptionally activate genes that mediate tumor progression and metastasis, including the RUNX2 target gene osteopontin (OPN/SPP1) in OS [270]. There is a correlation between high Wnt/β-catenin activation and RUNX2 expression in OS cell lines (Saos-2, MG63, U2OS, HOS, G292, 143B) [271].

miRNAs: Reduced expression of miR-338-3p was detected in MG63 and U2OS cells. RUNX2 and CDK4 are direct targets of miR-338-3p. The ability of miR-338-3p to inhibit MAPK pathway activation in MG63 cells has been demonstrated [272].

Low miR-203 expression was also observed in OS (MG63) cells. Suppression of miR-203 reduced the chemosensitivity of cisplatin in OS cells in vitro by targeting RUNX2 [273].

RUNX2 is a direct target gene for miR-320b. miR-320b acts as a tumor suppressor during OS invasion and migration by directly suppressing RUNX2 expression [274].

Low expression of miR-218 was observed in tumor tissues and OS cells. Overexpression of miR-218 suppressed the development and metastasis of U2OS cells. A targeted interaction between miR-218 and RUNX2 was detected (negative correlation in U2OS cells) [275].

#### 2.2.9. p53

The tumor suppressor protein p53 regulates the transcription of coding genes and the transcription of non-coding RNAs, especially miRNAs. The TP53 gene encodes the p53 protein, mutations of which have been associated with Li-Fraumeni syndrome and predisposition to the development of a number of tumors, including OS [276]. p53 controls apoptosis and cell growth by regulating bone homeostasis.

DNA damage induces phosphorylation of p53, allowing it to disengage from Mdm2, resulting in p53-mediated tumor suppression through cell cycle arrest or apoptosis. Mutation of the TP53 gene (tumor suppressor gene) is observed in more than 20% cases of OS [9].

miRNAs: The miR-34 family has been shown to induce G1 arrest and apoptosis in OS cells via its targets: CDK6, E2F3, cyclin E2, and BCL2 in a p53-dependent manner. Moreover, the expression of miR-34 was reduced in primary OS samples. miR-34 also inhibited p53-mediated cell cycle arrest and apoptosis in OS cells. In addition, p53 induces activation of miR-192,-194,-215 in U2OS cells carrying wild-type p53. Loss of miR-31 is associated with defects in the p53 signaling pathway, and overexpression of miR-31 inhibited proliferation of OS cell lines [9].

The evolutionarily conserved miR-34 family (a, b, c) is a tumor suppressor that responds to DNA damage and oncogenic stress and induces apoptosis and cell cycle arrest [276]. miR-34c regulates gene transcription in the p53-mediated cell cycle and apoptosis (CCNE2, E2F5, E2F2, HDAC1).

Epigenetic and genetic alterations associated with decreased expression of miR-34c in OS tissues (including cells with a high degree of 143B metastasis) are related to gene deletions and DNA hypermethylation [277]. Expression of miR-34c has been shown to be regulated by the p53 pathway.

Recent cell fate determination studies have integrated miRNAs as important modulators of osteoblast differentiation (miR-138, miR-206) [276].

Indirect reciprocal regulation was found between p53 and miR-203.

In OS tissues, low expression of miR-122-5p was detected against a background of low expression of its target (target gene), p53 (the lowest level was found in the U2OS cell line). Activation of miR-122-5p could inhibit proliferation and promote apoptosis of OS cells by inhibiting activation of the PI3K/Akt/mTOR pathway, which could be accompanied by targeted activation of p53 expression [278].

- FOXP1

FOXP1 plays an important role in cancer cell proliferation, growth, and metabolism [279]. FOXP1 promotes proliferation, tumor sphere formation, migration, invasion, and inhibition of anoikis. High expression of FOXP1 correlates with malignancies in OS cell lines and clinical biopsies. Mechanistically, FOXP1 interacts with p53 and suppresses its activity. It also represses transcription of p21 and RB. FOXP1 activators are ERK/JUN, c-JUN/c-FOS [280].

miRNAs: miR-181d-5p acts as a tumor suppressor in OS by targeting FOXP1 [281].

- Bak

Bax and Bak are members of the Bcl-2 family and important regulators of the intrinsic pathway of apoptosis. When exposed to apoptotic stimuli, both are not activated and oligomerize at the outer mitochondrial membrane, mediating their permeabilization, which is considered a key step in apoptosis [282]. Deregulation of the Bcl-2 family interaction network renders cancer cells resistant to apoptosis [283].

miRNAs: miR-342-5p post-transcriptionally inhibits BCL2L1 mRNA in SW1353 chondrosarcoma cells, while miR-491-5p inhibits epidermal growth factor receptor (EGFR) expression, whose spontaneous activation in the absence of ligand is associated with tumor progression. miR-491-5p also increases expression of the pro-apoptotic Bak protein. In addition, miR-342-5p has anti-apoptotic potential, leading to a decrease in the expression of pro-caspase-9 protein in chondrosarcoma cells [284]. In an in vitro study using OS cell lines (MG63, Saos-2, HOS), miR-342-5p and miR-491-5p showed significant anti-metabolic, cytotoxic, and pro-apoptotic effects [236].

It was found that the expression of miR-143 was significantly reduced in 143B OS cells compared with HOS cells (cells transformed with v-Ki-ras, which are characterized by a high rate of metastasis). Transfection of miR-143 resulted in reduced invasiveness and suppression of lung metastasis in vivo. This is supported by data showing that reduction of miR-143 in OS cell lines resulted in reduced viability and induction of apoptosis in a BCL-2-mediated manner [285]. It was found that 3-year, 4-year, and 5-year survival rates were significantly better in the group of patients with high expression of miR-143 (OS) [286]. In addition, MMP-13 was identified as one of the miR-143 target proteins by immunoprecipitation OS [287]. MMP-13 expression in tissue samples from OS patients with lung metastases was relatively low compared with patients without metastases.

- Bcl-xL

Bcl-xL is an anti-apoptotic protein [288] that regulates intrinsic apoptosis, mitochondrial bioenergetics, and oxidative stress. Bcl-xL maintains immune system functionality by mediating elimination of senescent cells [289].

miRNAs: The pathogenesis of OS is associated with high cell proliferation activity, the decrease of which under the influence of miR-133a also induces cell apoptosis. The oncogenicity of OS cell lines was reduced by miR-133-dependent downregulation of Bcl-xL expression [290].

Moreover, miR-377 is a tumor suppressor during OS progression, as evidenced by the inhibitory effect of miR-377 on tumor growth and the enhanced effect on cell apoptosis [291].

#### 2.2.10. VEGF-VEGFR-RAS-RAF-MEK-MAPK

VEGF, a critical factor in angiogenesis and vasculogenesis, plays an important role in the physiological vascular homeostasis of various tissues, as well as in the molecular pathogenesis of metastasis and tumor growth [292]. VEGF-A expression in OS has been associated with higher risk of lung metastases and lower survival [293]. VEGFR2 (vascular endothelial growth factor receptor 2), the major VEGF-A receptor involved in aniogenesis and vasculogenesis, and PD-L1, expressed in 64.5% and 35.5% of cells from OS, respectively, were associated with a prometastatic effect on lung and tumor growth [294,295].

miRNAs: In tissues from OS, a decrease in miR-150-5p expression was observed against a background of high VEGF expression. At the same time, overexpression of miR-150-5p significantly inhibited the proliferation and invasion of OS cells. Luciferase reporter gene analysis showed that VEGF is the target gene for miR-150-5p [296].

Compared with the normal osteoblast cell line (NHOst), low miR-1 expression was observed in OS tumor tissue and in the Saos-2 and U2OS cell lines against a background of increased VEGF expression. miR-1 acts as a tumor suppressor and potent inhibitor of OS cell proliferation, migration, and invasion by acting directly on the 3’UTR mRNA of a functional VEGF target gene (as determined by a dual-report luciferase assay) [297].

The OS samples showed low miR-134 expression. At the same time, overexpression of miR-134 inhibited tumor cell proliferation, migration, and invasion, and induced apoptosis in MG63 and Saos-2 cell lines. Luciferase reporter analysis confirmed that miR-134 mechanistically targets the 3’-UTR mRNA of VEGF and MYCN genes. Moreover, interferon regulatory factor (IRF1) is a key player in the control of miR-134 transcription by inhibiting cell proliferation, invasion, and migration in vitro and in vivo [298].

#### 2.2.11. PTEN

The phosphatase tensin homolog (PTEN) is a tumor suppressor gene that negatively regulates the PI3K/Akt/mTOR pathway [299]. A recent study identified four OS cell lines whose resistance to anlotinib was mediated by the loss of PTEN [300]. In addition, decreased expression of PTEN was observed in tumor samples from patients with OS and lung metastases [300].

miRNAs: According to the results of PCR-RT analysis, significant miR-1908 activation was detected in OS cells and tissues. At the same time, miR-1908 activation in OS tissues was significantly associated with cell proliferation, invasion, and tumor growth. miR-1908 directly inhibits PTEN expression [301].

The expression level of miR-221 is increased in OS tissues and cell lines. High expression of miR-221 was associated with increased ability of MG63 cells to proliferate, migrate, and invade. Suppression of miR-221 significantly increased PTEN expression, whereas an increase in miR-221 suppressed PTEN expression [302].

The direct target of miR-21 is PTEN, and activation of the PTEN/AKT pathway is observed in OS cells [303]. PTEN, a tumor suppressor gene, plays an important role in many types of solid tumors by modulating cell apoptosis and cell cycle, and its suppressor activity depends on the activity of lipid phosphatase, which negatively modulates the PI3K/Akt/mTOR pathway [303]. The expression of the tumor suppressor PTEN can be regulated by miR-21. Spry2 is an indirect target of miR-21, confirming the role of miR-21 in drug resistance in OS [304]. In addition, a correlation between miR-21 and FRS2, PIKFYVE, PIK3R1, and SOCS6 was detected [197].

In silico analysis showed that miR-187 targets PTEN in Saos-2 cells. Simultaneously, the suppression of miR-17 expression led to an increase in PTEN expression [305].

#### 2.2.12. Hedgehog (Hh)

Abnormal Hh signaling has a promigraphic effect and results in osteoblasts OS. However, in primary human OS samples and OS cell lines, activation of Hh signaling is detected [306].

miRNAs: miR-212 was found to contribute to proliferation and inhibition of apoptosis of OS cells by enhancing Hh signaling in the MG63 cell line [307].

#### 2.2.13. Additional Signallers in OS

- FOXK1

Suppression of FOXK1 reduced cell proliferation rate and development of malignant phenotype in human U2OS cells OS [308].

miRNAs: Downregulation of miR-186-5p was detected in OS tissues and cell lines, and negatively correlated with distant metastasis, Enneking stage and poor 5-year prognosis, and FOXK1 expression. Concurrently, overexpression of miR-186-5p attenuated OS cell proliferation, cell cycle, and EMT, and induced apoptosis [308].

- NOB1

The NIN1 (RPN12) homolog of binding protein 1 (NOB1) is located on chromosome 16q22.1 and consists of nine exons and eight introns [309]. The RNA substrate containing the D-site of proribosomal RNA is efficiently cleaved by NOB1 in a manganese-dependent manner, regulating protease function and RNA metabolism. NOB1 has been activated in various cancers and functions as an oncogene [310].

NOB1 is upregulated in OS tissues and cell lines and has been associated with poor survival in OS patients [311].

miRNAs: Low expression of miR-363 in OS tissues and cell lines was associated with low 5-year overall patient survival and was inversely proportional to tumor size, clinical stage, lymphoid metastases, and NOB1 expression. Overexpression of miR-363 inhibited proliferation, migration, and invasion by inducing apoptosis of MG63 cells. NOB1 is the target gene for miR-363 [311].

- TGIF2

TGIF2 acts as an oncogene. OS (MG63) cells express high levels of TGIF2 mRNA [312].

miRNAs: In silico analysis showed that miR-541 was associated with overall survival from OS [313]. miR-541 was significantly downregulated in OS tissues and cell lines, and miR-541 expression was strongly associated with tumor size, clinical stage, distant metastases, and tumor differentiation in OS patients. Survival curves showed that OS patients with low miR-541 expression had a poor prognosis, and Cox regression data indicated that miR-541 might be an independent predictor of overall survival in OS patients. At the same time, increasing miR-541 expression in OS cells by transfection resulted in inhibition of OS proliferation, migration, and invasion. TGIF2 is a potential target of miR-541 in OS cells [314]. In addition, TGIF2 is the target gene for miR-34 according to the results of experiments in nude mice (and MG63 cells) [315]. miR-129 is also a negative regulator of TGIF2 [312].

- RECK

Hypermethylation of the RECK motif promoter (a reversion-inducing cysteine-rich protein with Cazal motifs) is an important factor in the induction of the OS metastatic process [316].

miRNAs: RECK has been shown to be a direct target negatively regulated by miR-21 in the OS cell line and in human OS samples [317], and also inhibits invasion of OS cells by reducing the activity of matrix metalloproteinases [318]. miR-21 has been shown to be involved in the regulation of several tumor suppressor genes and apoptosis-associated proteins, including tropomyosin-1, programmed death protein 4, metalloproteinase-3 inhibitor, Fas ligand, heterogeneous protein K-RNA, and a phosphatase and tensin homolog (PTEN) 5. miR-21 may negatively regulate the expression of these genes and be involved in the growth of invasion and tumor metastasis.

RECK is a direct and functional target of miR-92b in OS [319].

- KLF10 (Krueppel-like factor 10)

KLF10 (Krueppel-like factor 10), originally named TGF-β-inducible early gene 1 (TIEG1), is a C2H2 zinc finger DNA-binding domain. By binding to Sp1 (specificity protein 1) sites on DNA and interacting with other regulatory transcription factors, KLF10 stimulates and represses the expression of many genes. KLF10 plays the role of a tumor suppressor. KLF10 has an antiproliferative effect and induces apoptosis in carcinoma cells [320].

miRNAs: High expression of circ-0003998 was observed in OS tissue samples and OS cell lines. Circ-0003998 can bind miR-197-3p and increase KLF10 expression in MG63 and Saos-2 cell lines, promoting OS growth, invasion, and progression. Suppression of circ-0003998 decreased the proliferative and invasive capacity of OS cells in vitro [321].

- HDAC1

A preliminary study has shown that histone deacetylase 1 (HDAC1) and SP1 are activated in tissues from OS and promote tumor progression [322]. There is also a positive feedback loop between activating transcription factor 4 (ATF4), which can activate the processes of proliferation and apoptosis, and histone deacetylase metastasis associated protein (MTA1)/HDAC1 [323]. ATF4 promotes OS growth and metastasis, and MTA1 stabilizes ATF4 by recruiting HDAC1, while ATF4 transcriptionally activates MTA1 expression in OS [323].

miRNAs: miR-326 has been found to be involved in the pathogenesis of OS and is a potential diagnostic and prognostic marker for OS, whose decreasing expression indicates distant metastasis and late clinical cancer stage [324]. miR-326 is negatively regulated by Sp1, which recruits HDAC1 [322]. Selective inhibition of HDAC1 prevents tumor growth and metastasis in OS.

miR-449a downregulates HDAC1 expression by regulating histone acetylation and promoting differentiation of induced human pluripotent cells (iPS) into osteoblasts. This process was accompanied by a decrease in HDAC1 expression and a higher level of histone acetylation, particularly in the Runx2 promoter [325]. The role of miR-449a in the pathogenesis of OS is well established. Given the antitumor potential of miR-449a and its low expression in OS, the level of this miR could be a diagnostic and prognostic indicator. Moreover, the downstream targets of miR-449a in OS cells are the Zeste Homologue 2 (EZH2) enhancer and the phosphatidylinositol 3-kinase (PI3K)/protein kinase B (Akt) pathway involved in epithelial-mesenchymal transition. In vivo studies have confirmed that local upregulation of miR-449a expression in OS cells leads to tumor growth arrest [326].

miR-874-3p is known to induce osteoblast differentiation and mineralization by inhibiting Hdac1 expression and enhancing Runx2 transcriptional activation [327].

- HDAC4

HDAC4 induces proliferation, migration, and invasion of OS cells [328].

miRNAs: Overexpression of miR-140 is associated with chemoresistance to methotrexate and 5-fluorouracil in OS xenograft models, suppresses cell proliferation, and induces G1 and G2 arrest in U2OS and MG63 cells OS. Moreover, miR-140 can negatively regulate histone deacetylase 4, which interacts with the expression of p21, resulting in resistance to 5-fluorouracil. In addition, miR-215 decreased cell proliferation and induced G2 arrest and increased chemoresistance to mettrexate in U2OS cells [329]. HDAC4 is the downstream target gene of miR-637 [328].

- SND1

The staphylococcal nuclease Tudor (SND1) is an essential component of the RNA-induced silencing complex (RISC) and an important component of the RNA interference mechanism (RNAi). SND1 is a highly conserved component of the programmed cell death degradation group [330]. SND1 has been shown to play an important role in the initiation, development, and metastasis of various cancers. A study of patients with OS identified a set of single nucleotide polymorphisms associated with a predisposition to OS localized in the gene involved in RISC components (CNOT1, CNOT4, SND1), indicating the role of SND1 in the development of OS [331].

miRNAs: Low expression of miR-296-5p against a background of high SND1 expression was detected in OS cell lines (HOS, U2OS, Saos-2, MG63) compared with healthy bone tissue. SND1 is a target for miR-296-5p. Increasing the level of miR-296-5p in OS cells may serve as an effective strategy to treat OS [332].

- Foxp4

The forkhead-box family (FOX) comprises a group of transcription factors involved in cell- and tissue-specific gene regulation. Forkhead-box P4 (FOXP4) is a member of the FOXP subfamily of winged helix transcription factors and encodes a protein of 685 amino acids. FOXP4 is expressed mainly in intestinal, neural, and lung tissues during embryonic development. In addition, FOXP4 is required for T lymphocyte development and antigenic response [333]. A significant increase in the expression of the FOXP4 gene and protein in tissues and OS cell lines was detected [334].

miRNAs: Low levels of miR-491-5p have been shown to promote human OS cell proliferation via the FOXP4 pathway in vitro. Increasing the expression level of miR-491-5p contributed to the inhibition of OS cell proliferation and increased the rate of apoptosis by targeting FOXP4 [334].

- GPX4

Glutathione peroxidase 4 (GPX4) maintains cellular redox homeostasis [335].

miRNAs: Expression of miR-1287-5p was suppressed in human OS but increased upon ferroptotic stimulation. Mechanistically, miR-1287-5p directly binds to the 3’-untranslated region of glutathione peroxidase 4 (GPX4), and inhibits the amount and activity of its protein. It was found that a miR-1287-5p mimetic significantly increased the sensitivity of human OS cells to cisplatin chemotherapy [336].

## 3. Heterogeneity of the Cellular Component of the OS Substrate

OS is a malignant tumor whose progenitor cells may be osteogenic lineages derived from MSCs or undifferentiated MSCs themselves under the influence of relevant carcinogenic factors and epigenetic effects of the tumor microenvironment [32].

Oncogenesis and tumor progression are supported by reciprocal signaling between OS cells and the tumor microenvironment (TME)/bone microenvironment (BME), including the extracellular matrix and cellular components (stromal cells, immune cells, macrophages, lymphocytes), endothelial and bone cells (osteoblasts, osteoclasts, osteocytes), including MSCs, pericytes, and fibroblasts [337]. In general, TME of primary tumors (in children) remains relatively unexplored [35]. The functional potential of non-malignant cells in mesenchymal tumors in children is not well characterized [35]. Despite the importance of the TME in various types of solid tumors, the study of the interaction processes between stromal and tumor cells OS has not received due attention, which is reflected in a lack of clear understanding of the TME and its role in the development and progression of OS [338]. The bone marrow is a specialized microenvironment that contains heterogeneous cell types. Therefore, identifying the full spectrum and mechanisms of the interaction of cells involved in the initiation/maintenance/development of OS will allow identification of new targeted therapeutic targets.

At the same time, OS is a heterogeneous tumor containing cells at different stages of differentiation during ontogenesis [29].

It has been established that the immunological microenvironment of the tumor (TIME) plays an important role in the development and progression of OS [45,339]. There is intratumoral heterogeneity among immune cells in OS [12].

According to single-cell RNA sequencing (scRNA-seq) results, new cell populations responsible for intratumoral heterogeneity in OS have been identified [340]. For example, in samples from osteoblastic OS, an increase in mesenchymal and myeloid cells was detected against a background of a decrease in T and B lymphocytes. The studied cells were classified into six cell lines with 29 cell clusters based on unique molecular identifiers, including expression of specific transcription factors and genes (supplemented by analysis of target databases and information from the Gene Expression Omnibus GEO, http://www.ncbi.nlm.nih.gov/geo/ (accessed on 1 March 2023) database numbered GSE21257, GSE3905, GSE16091) [341]:

- Mesenchymal;
- T lymphocytes (Treg, CD4+, CD8+ (highly malignant), NKT, DK, plasmacytoid DK-pDK) [341];
- B lymphocytes (naive B cells, activated B cells, plasma cells);
- Myeloid cells (mast cells, monocytes, M1, M2 macrophages, tumor-associated macrophages (TAM));
- Osteoclasts;
- Endovascular (endothelial, parietal) cells.

The pool of mesenchymal cells included BM-MSCs, osteoblasts, adipocytes, chondroblasts, and seven OS tumor cell clusters: CSC-like cancer cells, OS-A1, OS-A2, OS-B1, OS-B2, OS-C1, and OS-C2 [340]. It is possible that different subtypes of OS cells overlap in their functional (genetic, morphological) potential (aggressiveness and proliferation kinetics) with known in vitro models, and OS cell lines (Saos-2, MG63, HOS) [342]. Interestingly, the SaSO-2 cells show a phenotype that resembles normal mesenchymal progenitor cells. In turn, the extremely aggressive HOS cells exhibit an apparent hexagonal morphology that resembles epithelial cells. MG63 cells exhibit an intermediate phenotype between Saos2 and HOS cells [26].

The CSC-like cells comprised four subclusters: CSC, CSCL1, CSCL2, and CSCL3 [340]. Stem cell markers were strongly expressed in BM-MSC and CSC-like clusters and partially expressed in OS-A1/A2/B1/B2. At the same time, markers for chondrocytes and adipocytes were expressed at low levels in all clusters of OS. Differentiation of the line OS-A was associated with the expression of the genes MYC and CYR61; the line OS-B with the expression of the gene CDK4; in the cell line OS-C, a gradual increase in the expression of the genes TIMP3 and MMP13 was observed. CSC cells expressed more osteoblastic markers (genes) COL1A1, COL1A2, and SPARC compared to BM-MSC.

In contrast to osteoblasts, overexpression of genes associated with tumor progression (SPP1, SERPINA1) was observed in CSC-like cells. In addition, CSC-like cells highly expressed drug resistance genes: FTL, XIST, and MT1G [340]. The authors noted that gene markers of CSCL1/2/3 were partially similar to the clusters OS-A2/B2/C2 (MT1G, SERPINA1 in CSCL1; SPP1, MMP13 in CSCL3), suggesting that CSCL1/2/3 may be precursors of OS-A2/B2/C2 [340].

In addition to the cell subpopulations described, MSCs from bone marrow and adipose tissue (hASCs) [27,343], which are involved in cell proliferation, progression, and metastasis, are an important component of the OS tumor microenvironment [344].

The cellular composition of OS described above supports the notion of a hierarchical organization of sarcoma and subpopulations of self-renewing cells that can generate a complete repertoire of tumor cells and exhibit features of tumor re-initiation upon relapse [32].

### 3.1. Characteristics of the Tumor Microenvironment and the Behavior of MSCs in OS

Mesenchymal stromal cells (stem cells) (MSCs) are highly mobile and capable of migrating throughout the body to repair damaged tissues. This is an extremely useful property of MSCs as a tool for regenerative medicine [345]. On the other hand, the mesenchymal phenotype can be reactivated in cancer cells, making malignant epithelial cells motile and invasive through the epithelial-mesenchymal transition (EMT), which increases tumor aggressiveness, including the ability to sustain proliferative signals, evade growth suppressors, resist cell death, maintain replicative immortality, induce angiogenesis, activate invasion, and metastasis [26].

In human carcinomas and sarcomas, a combination of differentiated tumor cells, cancer stem cells (CSCs), cancer-associated fibroblasts (CAFs), mesenchymal stromal cells (MSCs), and immune cells form a tumor volume in which an immunosuppressive microenvironment [346] stimulates tumor growth and metastasis [347].

Myeloid-derived suppressor cells (MDSCs) are a heterogeneous population of immature myeloid cells (IMCs) that can strongly infiltrate osteosarcoma tissue and inhibit the expansion of cytotoxic T cells [348]. Currently, there are no specific markers to identify MDSCs. At the same time, MDSCs have been shown to be able to interact closely with osteoclasts, osteoblasts, chondrocytes, and other stromal cells in the tumor microenvironment of osteosarcoma, which may contribute to poor prognosis. In addition, their role in osteoregeneration is discussed, making these cells potential and promising targets for targeted therapy [349].

Classically, MSCs play their essential role mainly through mechanisms dependent on cell contacts and soluble factors [350].

### 3.2. MSC Migration and OS Growth

The tumor microenvironment contains malignant cells and healthy stromal, vascular, and immune cells (as described above). Activated stromal cells have been found to mix with tumor cells, immune cells, and other cell types in the pseudocapsule surrounding the tumor, and to support endothelial tube formation during angiogenesis [35]. Structural ECM proteins (collagens) and cellular matrix proteins (osteopontin) support and signal cell movement. Simultaneously, single cell and cell chain migration of mesenchymal sarcoma cells are involved and regulated by the state of the ECM, including integrins (cadherins) and proteases [35].

MSCs within the TME play an important role in the pathogenesis of OS, from stem cells to cells responsible for the growth, progression, metastasis, and drug resistance of OS [351]. At the same time, the stage of MSC differentiation influences the OS phenotype [351]. The HIF pathway induces the secretion of angiogenic factors by tumor cells and promotes MSC migration into tumor tissue, metastasis, and tumor progression [352]. Lin et al. found that hMSC-derived microvesicles (MVs) promoted the proliferation and migration of U2OS cells under hypoxia in vitro, which was partially associated with the PI3K/AKT and HIF-1α signaling pathways. MVs co-injected with U2OS cells promoted tumor growth in a mouse xenograft model in vivo. SiHIF-1α transfection reversed these changes to some extent [353].

Thus, cross-talk between migrated MSCs and OS cells is largely supported by cytokines/chemokines and exosomes (extracellular vesicles) [354]. For example, transforming growth factor β (TGF-β), stroma-derived factor (SDF1), and osteoprotegerin (OPG) (activates the SDF-1/CXCR4 axis) [355], activated plasminogen, the progelatinases MMP-1/9, MIF, and IL-6 induce migration of MSC to OS cells and promote tumor growth and metastasis through neovascularization [352]. MCP-1, GRO-α, and TGF-β1 also induce migration (tropism) from BM-MSC to OS cells (Saos-2, MG63, and HOS) [342]. At the same time, exosomes secreted by MSCs (MSC-derived exosomes) contain stimulatory factors (for example, microRNA-208a) [356] or growth inhibitory factors (microRNA-150) and OS progression [357].

### 3.3. Transdifferentiation/Plasticity of MSC

The problem in sarcomas lies in the fuzzy differences in morphological and functional characteristics (features) between malignant cells and healthy stromal cells due to their common mesenchymal origin. In this case, cellular transdifferentiation of stem cells from bone marrow may occur, which is important for the progression of OS [342]. Hypoxic conditions in the TME stimulate anaerobic glycolysis, leading to acidification of the extracellular matrix (ECM), which can promote conversion of normal MSCs (CD105+CD73+CD90+CD29+CD44+CD79a-CD11b-CD19- or CD45-CD34-CD14- HLA-DR-) [13] in tumor cells through activation of the NF-kB pathway [358] and expression of CCL5, CXCL5, CXCL1, IL-6, IL-8, and CXCR4. In addition, conversion of MSCs to OS cells is favored by abnormal expression of Rb, c-Myc, TP53, K-Ras, and IHH genes [359].

MSCs can produce soluble factors that regulate cancer cell stemness [360]. Specifically, MSCs secrete TGF-β and IL-6, which increase the stemness, cell proliferation, migration, and metastatic potential of CSCs (OS) [361]. Hypoxia is also a trigger for increased expression of TGF-β, which promotes the formation of CSC OS from MSCs [362].

It has been found that BM-MSCs interacting with cancer cells transdifferentiate into tumor stromal cells, including pericytes, endothelial cells, and cancer-associated fibroblasts [342].

#### 3.3.1. Cancer-Associated Stromal Fibroblasts (CAFs)

Cancer-associated fibroblasts (CAFs) are the most abundant cells in the TME [363] and can lead to cancer progression by influencing the malignant potential of tumor cells (cancer stem cell turnover, proliferation, motility). Drug resistance and the epithelial-mesenchymal transition (EMT) [364,365,366] reduce the efficacy of immunotherapy [367]. Differentiation of BM-MSC into CAF is a multistep biological process [368] that results in cells that are functionally and phenotypically distinct from normal tissue fibroblasts [369].

Activated CAFs are spindle-shaped, associated with round nucleoli and myofilaments, and express markers such as SMA, FAP, FSP, S100A4, PDGGFR α/β, and tenascin-C [370,371]. It has been shown that OS CAF forms three separate subclusters with novel gene markers and functions [372,373].

The conversion of MSC to CAF occurs with a decrease in pH in the ECM and is dependent on GPR68, a G protein-coupled receptor and YAP. The induction of the transition from BM-MSC to CAF in OS is related to the IL-6/STAT3, Notch, Akt signaling cascades [352]. Thus, the conditioned medium of the OS U2OS cell line (BM-U2OS) induced the differentiation of BM-MSC into CAF by upregulating IL-6/STAT3 expression, and promoted the mobility and invasiveness of OS cells [374]. Transdifferentiation of BM-MSCs into myofibroblastic subsets of CAF, expressing α-SMA and collagen I-α1, can be induced by contact with OS cells and increased levels of the cytokines/chemokines MCP-1, GRO-3a, IL-6, and IL-8 in the TME [13,342].

Direct exposure of OS cells to HBMSCs (co-culture system) induces Notch-mediated activation of Akt signaling, which induces HBMSCs (BM-MSCs) to differentiate into CAF (by a novel mechanism) [375].

One of the pathways by which CAFs regulate the invasion and migration ability of OS cells is the release of the exosomal miR-1228 CAF, which represses mRNA expression of the SCAI gene [371]. In addition, CAFs are able to regulate EMT and the recurrence of OS through secretion of the extracellular matrix enzyme lysyl oxidase (LOX), whose substrates are collagen and elastin. LOX acts on VEGF induction and HIF1α activation, and promotes tumor progression and metastasis [376,377], including OS [378].

In addition, high expression of the cell surface glycoprotein extracellular matrix inducer (EMMPRIN) in OS (Saos-2) and CAF cells regulates VEGF and MMP production, and mediates metastasis [379]. It was found that the CAF marker is the fibroblast activation protein (FAP), which induces phosphorylation of AKT and ERK, and activates angiogenesis via VEGF in MG63, U2OS, and HOS cells [380].

#### 3.3.2. Cancer Stem Cells (CSCs) and MSCs

Cancer stem cells (CSCs) are a small subset of tumor cells with stem cell properties; one of the proposed criteria for their definition is expressing the transcription factors Nanog, Oct3/4, and Sox2 [381]. They are capable of self-renewal and differentiation into various cell types and are responsible for tumorigenesis, local and systemic recurrence, and resistance to chemotherapy or radiotherapy in solid malignancies [382]. CNSs have been shown to include tumor-initiating cells and drug-resistant cells, as well as cells with high proliferative capacity and cells in the quiescent state (which are the most difficult to identify) [383]. In addition, putative CSC phenotypes may differ depending on the different methods of cell isolation [384].

CSC-like chemoresistant cells were identified in OS [385]. Gibbs et al. first confirmed the existence of CSCs in OS [386]. The theory of primary CSCs is that CSCs undergo symmetric or asymmetric division [361], are capable of regenerating the entire tumor mass after chemo/radiotherapy, and lead to recurrence and metastasis (particularly through activation of lysine oxidase (LOX) in a hypoxic environment) [387,388]. Clinical cases indicate the persistence of cancer cells that remain immobile at the site of the primary tumor during the remission phase and are reactivated by a change in the local microenvironment [389].

Osteosarcoma-derived CSCs and CSC-like cells express CD24, CD177, Stro-1 (IBSP), CD133, the detoxifying enzyme aldehyde dehydrogenase-1 (ALDH1) [387,390], CD273 [19], and CD271 [391]. CD271 is known to be a receptor for neural crest growth factor and is the marker for bone marrow-derived MSCs [391]. OS-CSCs are characterized by drug resistance associated with high DNA repair capacity, inhibition of apoptotic signaling, increased lysosomal activity due to overexpression of vacuolar ATP, and enhanced drug efflux due to overexpression of ABC transporters [10,32,392].

The niche of CSCs is considered a separate component of the TME that supports CSC self-renewal and survival. The tumor niche also includes functionally heterogeneous stromal cells, extracellular matrix proteins, and signaling factors located near the CSC. The mechanical properties of the ECM (extracellular matrix) (stiffness, topological alignment) and hypoxia may induce different phenotypes of CSCs. Moreover, the ECM associated with CSCs promotes their self-renewal and survival [393]. It is noted that minimal residual disease is defined as the presence of treatment-resistant malignant cells (persistent, quiescent, dormant cancer cells in residual tumors, including circulating tumor cells and disseminated tumor cells in bone marrow and metastatic foci) that remain in the body after remission and lead to recurrence/metastasis [387,394,395,396].

Tumor-initiating cells (TICs) with stem cell properties have been identified using the mesenchymal markers CD117 and Stro-1 in humans and mice with increased metastasis and drug resistance OS [397]. Other authors consider TICs as CSCs [381].

At the same time, according to the hypothesis of Brune JC et al. [398], BM-MSCs can become tumor stromal cells through epigenetic stress and aberrant differentiation [62,347].

Mesenchymal stem cells migrating to the tumor site (or BM-MSC) under the influence of the tumor microenvironment (TME) acquire a new phenotypic profile and transform into CAF or CSC. At the same time, three or more cell subtypes have been identified in the pool of CAF and CSC. CAF contribute to the progression and metastasis of OS. Several CSC subtypes are thought to be able to migrate to malignant OS cells and form a (new) tumor site. On the other hand, healthy MSCs are able to inhibit the development of OSCs by inducing apoptosis (upregulation of Bax expression, downregulation of Bcl2, survivin) and autophagy (ATG5, ATG7, Beclin1).

### 3.4. Transformation of MSCs into OS Cells

In mouse models, OS genetic loss of the cdkn2 gene locus in MSCs was associated with their tumor transformation [399]. At the same time, transformation of human MSCs into OS was observed against a background of simultaneous overexpression of the c-Myc gene and silencing of the Rb gene [400]. In addition, aneuploidy (change in chromosome number) and loss of genome can transform MSCs into OS cells [399]. Members of the activator-1 protein family have been shown to induce osteosarcomogenesis in immortalized hMSCs. Overexpression of c-JUN or c-JUN/c-FOS acts as an oncogenic factor and generates OS with an osteoblastic or pleiomorphic osteoblastic phenotype [401].

Bidirectional contact between MSCs migrating into OS and tumor cells has been found to affect transdifferentiation (plasticity) of both healthy and malignant cells. According to one study [342], the interaction between BM-MSC and OS cells (Saos-2, MG63, HOS) promotes plasticity of tumor cells toward an amoeboid phenotype, and influences transendothelial cell migration and subsequent metastasis. Moreover, the interaction between BM-MSC and OS cells enhances chemoattraction and invasiveness of endothelial cells and stimulates tumor vascularization by forming a new capillary network [342]. In turn, MSCs are recruited from normal tissue into TMEs and adopt a protumor phenotype. Thus, MSCs from both normal and tumor tissues can accelerate the growth of OS [402]. At the same time, MSCs from OS tissue are more potent effectors of tumor progression [350].

### 3.5. Involvement of MSCs in Metastasis of OS Cells

Metastasis is associated with changes in the phenotypic activity of tumor cells and the tumor microenvironment. For example, downregulation of E-cadherin occurs during the cellular epithelial-mesenchymal transition (EMT) program, and activation of E-cadherin is associated with mesenchymal-epithelial transition (MET) during the development of distant metastases [35]. The mesenchymal-amoeboid transition (MAT) is observed in OS during transendothelial migration [342]. Endothelial cells and pericytes are thought to be sources of Notch pathway activation in OS.

BM-MSCs generally promote invasiveness and transendothelial migration of OS cells through the mesenchymal transition to the amoeboid state [342]. At the same time, tumor-activated MSCs contribute to stem cell formation and migration potential of OS through secretion of IL-6 [347]. Recently, multipotent adipose-derived MSCs (hAD-MSCs) were shown to be potential promoters of OS progression and to significantly affect the tumor microenvironment [28,403]. Thus, a unique clinical case of OS recurrence after autologous transplantation of adipose tissue (with MSC) (lipofilling) was described [17]. However, Hernigou et al. [20] reported that autologous adjuvant therapy with concentrated bone marrow cells did not increase the risk of tumor recurrence compared with controls in 92 individuals treated after bone tumor resection.

Human MSCs injected intravenously into mice with Saos-2 OS cells caused tumor expansion and resulted in severe osteolytic lesions and a high rate of lung metastases [18]. In another study, intravenous administration of MSC also induced the development of OS lung metastases [404]. At the same time, local injection of MSC into the area of surgically removed OS did not result in the development of lung metastases or local recurrence in mice. According to [404], direct injection of MSC into the primary OS node in mice suppressed tumor recurrence and inhibited the growth of remaining OS cells; in contrast, intravenous injection of MSC promoted lung metastasis.

### 3.6. MSC and Tumor Cell Death

CXCR1 (chemokine receptor 1 with CXC motif)/Akt activation induced by IL-8 secreted by MSC promotes resistance of OS cells to anoikis (programmed cell death by loss of contact between the extracellular matrix and other cells) [405] and lung metastasis [406].

Wharton jelly-derived MSCs (WJ-MSC), when cultured with OS cells, resulted in inhibition of OS cell proliferation and migration with an increase in the pro-apoptotic transcription factor BAX, the suppression of the anti-apoptotic transcription factors BCL2, and SURVIVIN, and the activation of the autophagy genes ATG5, ATG7, BECLIN1 in MG63 cells [407].

Epigenetic changes in MSCs caused by expression and secretion of the proangiogenic and anti-apoptotic factors IGF1 and leptin resulted in decreased staurosporine-induced cell death in the OS cell line [404,408,409], indicating a relationship effect between MSC and the administered dose: low concentrations of AD-MSC had an inhibitory effect, while higher concentrations had a stimulatory effect on the growth of OS.

Thus, there is a dual role for MSCs in the development of OS, which currently makes their use in regenerative therapy in tumor patients difficult. On the one hand, MSCs are involved in tissue regeneration processes, and on the other hand, they are the main regulatory cells of oncogenic processes [249,410,411]. MSCs modulate the growth, development, progression, metastasis, and drug resistance of OS. At the same time, MSCs have both pro- and antitumor potential [412,413,414], and the detailed mechanisms of their modulatory effects remain unexplored [350].

## 4. The Potential of MSCs in OS Therapy

MSCs are being extensively studied for their use in the treatment of OS. MSCs are considered attractive cells that can form the basis of (and enhance and refine) novel therapeutic antitumor approaches based on local cell-based delivery of drugs, biological molecules, or genes [415], as well as the regulation of signaling pathways and secretory potential of MSCs [359]. Detailed molecular interpretation of the mechanisms by which signaling pathways and miRNAs interact will complement promising molecular studies of targeted OS therapy [416,417,418,419,420,421,422,423,424,425,426].

Research has shown that MSCs can be used as carriers of cancer genes in malignant tumors that can inhibit tumor growth or metastasis. For example, culturing human BM-MSCs expressing cytosine deaminases/5-fluorocytosine (CD/5-FC) with the human OS cell line Cal72 suppressed tumor cell growth, which was confirmed by in vivo experiments in mice [415]. MSCs transfected with the osteoprotegerin gene (OPG) can migrate to OS after intravenous injection and produce OPG, reducing tumor volume and bone loss in vivo. However, some MSC-bearing OPGs were retained in lung tissue [427].

Apoptosis-inducing TNF ligand (TRAIL) delivered by AD-MSC has been shown to have an antitumor effect (OS) through a longer half-life and the secretion of synergistic factors [428,429].

MSCs can be used to deliver anticancer drugs, particularly nanoparticles and photosensitizers that cause OS cell death in vitro due to photoactivation by the release of reactive oxygen species (ROS). The efficacy of photoactivation of nanoparticle-loaded MSCs in vitro has been demonstrated in an ectopic model of OS in mice [430]. However, anticancer agents used in carrier systems for MSCs can kill the MSCs themselves [431].

MSCs can carry oncolytic adenoviruses and granulocyte colony-stimulating factor, resulting in increased immune infiltration and decreased growth OS [432]. However, the pharmacokinetic properties of MSC vectors still require extensive research to ensure not only efficacy but also safety [13].

Despite promising advances in the use of MSCs for anticancer drug delivery and regeneration of bone defects after OS resection, there are concerns about their side effects (acceleration of cancer cell proliferation, induction of lung metastases, chemoresistance to doxorubicin and cisplatin) that require further research [350].

Due to the existing risk of tumor transformation of MSCs and stimulation of OS growth, another direction in OS therapy is the use of exosomes isolated from MSCs. Exosomes are excellent nanocarriers for specific and efficient delivery of chemotherapeutics and miRNAs. In particular, exosomes isolated from MSCs migrated to the tumor through chemotaxis via the SDF1-CXCR4 axis [433]. Exo-Dox (derived from BM-MSC) showed high cell uptake efficiency and antitumor activity in the OS MG63 cell line, and low cytotoxicity in the H9C2 cardiomyocyte line [434].

In general, the processes of OS initiation, development, progression, metastasis, and drug resistance are regulated by different signaling pathways that interact and overlap with each other, and which are also controlled by different miRNAs (Table 1). Individual miRNAs in OS have a direct effect on components of a particular signaling pathway and may act as a link between multiple signaling cascades. The use of methods to locally modulate the expression of signaling pathways and genes/molecules responsible for growth and progression, especially pro- and anti-oncogenic miRNAs in the OS tissues themselves, and their delivery based on modified MSCs and their exosomes, gives us hope for the development of effective targeted cell-based methods to combat OS.

BM-MSC-derived extracellular vesicles have been shown to inhibit the malignant behavior of OS cells [414]. Exosomes (EVs) derived from AD-MSC and enriched in miR-101 can suppress OS by inhibiting the activity of the proto-oncogene BCL6, enabling innovative therapy for metastatic OS [420]. Similarly, EVs derived from MSCs and carrying miR-150 suppress proliferation and migration of OS cells by targeting IGF2BP1 (insulin-like growth factor 2 mRNA binding protein 1) [357].

Synthetic introduction of miR-143 into MSCs increases secretion of ecosomal miR-143 in a condensed medium and enables suppression of OS cell migration by rapid entry of miR-143-containing exosomes into recipient cells [435]. miR-206 can inhibit OS progression [436,437]. A conditioned environment containing GW4869 (a neuronal noncompetitive inhibitor of sphingomyelinase) is a prerequisite for high miR-206 levels in BM-MSCs. The miR-206-containing vesicles inhibited the ERK1/2-Bcl-xL pathway, suppressing the malignant behavior of OS cells [438]. In addition, miR-101, present in extracellular vesicles, acts as a tumor suppressor in OS [420] by inactivating the ROCK signaling pathway (Table 1).

Cancer vaccines and immunotherapies based on exosomes are a current area of molecular therapy for cancer and are being tested in clinical trials [439]. There are a number of clinical trials investigating the anticancer effects of MSC-derived exosomes in various cancers (NCT01159288, NCT03608631). Active development in this area of medicine may anticipate the early use of such constructs in OS combination therapy.

However, data on the in vivo use of miRNAs in exosomes/extracellular vesicles are still extremely sparse. For example, the antitumor efficacy of MSC-exo-miR-150 has been demonstrated in vivo; however, the safety of this construct has not been adequately investigated [357]. However, the therapeutic application of targeted delivery of miRNAs via modified MSCs undoubtedly has great therapeutic potential, especially for the treatment of OS.

## 5. Conclusions

OS is an aggressive tumor characterized by rapid growth and progression, cell cycle disruption, and high metastatic activity. The major challenges in OS therapy are its heterogeneity and drug resistance. The development of new therapeutic approaches to overcome the major growth mechanisms of OS is urgently needed.

MSCs are important cells that have received considerable attention in cancer research. Currently, new cell-based methods for using MSCs in medicine are being actively investigated and tested, especially as carriers for chemotherapeutics, nanoparticles, and photosensitizers. However, despite the inexhaustible regenerative potential and known anticancer properties of MSCs, they may trigger the development and progression of bone tumors.

**Figure 1:**
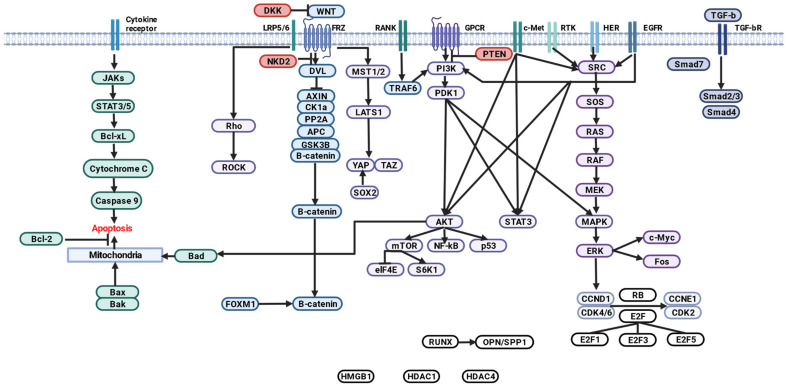
Signaling pathways involved in the pathogenesis of osteosarcoma.

**Figure 2:**
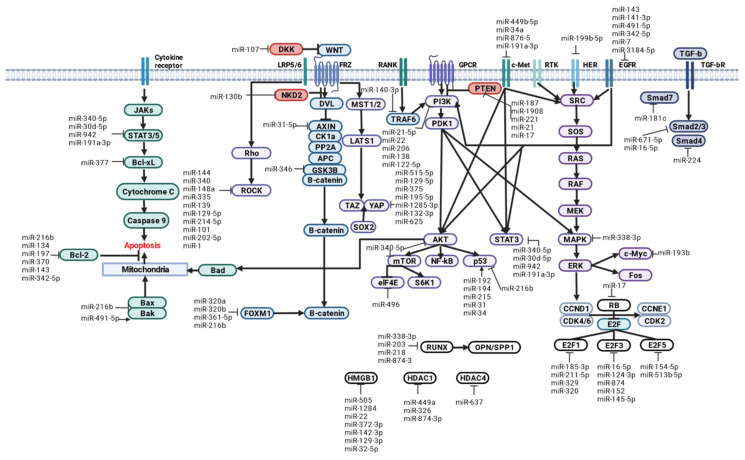
Major signaling pathways and regulatory miRs involved in the pathogenesis of osteosarcoma.

**Figure 3:**
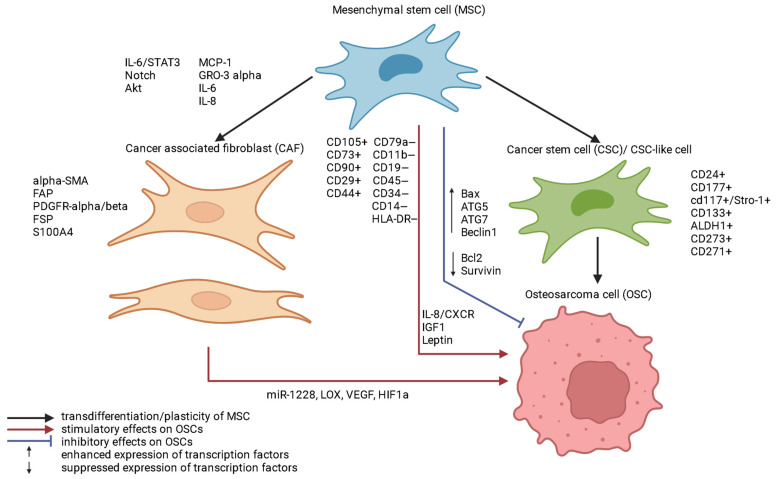
Dual role of MSCs in osteosarcoma according to the literature.

In our opinion, the current research focus may be on the use of methods that promote programmed, time-dependent death of modified MSCs after administration of a therapeutic agent to the tumor site, and preclude their subsequent tumor transformation [440]. A very promising and actively developing direction is the use of MSC exosomes for local delivery of drugs, genes, and various biological molecules [13]. In this regard, deciphering the signaling pathways associated with the pathogenesis of OS (Figure 1), elucidating the role of epigenetic factors, particularly miRNAs, in regulating the growth of OS (Table 1 and Figure 2), and understanding the mechanisms of the unclear interaction between OS cells and MSCs (Figure 3) will contribute to the advancement of cellular and molecular methods to combat OS to reduce the growth, development, metastasis, and drug resistance of malignant cells.

## Figures and Tables

**Table 1:** Signaling pathways and regulatory miRNAs involved in the molecular control of osteosarcoma growth.

Effects	Signal Path	Component of the Signal Path	Activation (↑)/Inactivation (↓) of the Signal Path Component in OS	miRs Regulating Components of the Signaling Pathway	High (↑)/Low (↓) miRs Expression Levels in OS	Influence of miRs on Components of Signaling Pathways (Suppression ↓/Mediated Activation ↑)	References
Proliferation, migration, invasion		NKD2	↓	miR-130b	↑	↓	[83]
		GSK-3β	↓	miR-346	↑	↓	[84]
		TCF-4	↑	miR-4695-5p	↓	↓	[87]
		TCF-7	↑	miR-192	↓	↓	[87]
		FOXM1	↑	miR-320a/b	↓	↓	[91,92,93]
		FOXM1	↑	miR-361 miR-361-5p	↓	↓	[94,95]
		FOXM1	↑	miR-216b	↓	↓	[96]
Apoptosis		Bax/Bcl-2	↓/↑	miR-216b	↓	↑/↓	[96]
		FOXM1	↑	miR-134	↓	↓	[97,98]
Cell cycle		E2F1	↑	miR-185-3p	↓	↓	[101]
EMT		E2F1	↑	miR-211-5p	↓	↓	[101]
		E2F1	↑	miR-329	↓	↓	[102]
		E2F1	↑	miR-320	↓	↓	[103]
		E2F3	↑	miR-145-5p	↑	↑	[108]
		E2F3	↑	miR-16-5p	↓	↓	[109]
		E2F3	↑	miR-124-3p	↓	↓	[110]
		E2F3	↑	miR-874	↓	↓	[111]
		E2F3	↑	miR-152	↓	↓	[112]
		E2F5	↑	miR-154-5p	↓	↓	[105]
		E2F5	↑	miR-513b-5p	↓	↓	[106]
Cell cycle		RB1	↓	miR-17	↑	↓	[116,117]
Chemoresistance, EMT		NRF2	↑	miR-340-5p	↓	↓	[120]
Migration, invasion, stemness		YAP	↑	miR-515-5p	↓	↓	[124]
		YAP	↑	miR-129-5p	↓	↓	[125]
		YAP	↑	miR-375	↓	↓	[126]
		YAP	↑	miR-195-5p	↓	↓	[127]
		YAP	↑	miR-1285-3p	↓	↓	[128]
		YAP	↑	miR-132-3p	↓	↓	[129]
		YAP	↑	miR-625	↓	↓	[130]
		ROCK	↑	miR-144	↓	↓	[131,137]
		ROCK1	↑	miR-340	↓	↓	[134]
		ROCK1	↑	miR-148a	↓	↓	[139]
		ROCK1	↑	miR-139	↓	↓	[140]
		ROCK1	↑	miR-335	↓	↓	[141,142]
		ROCK1	↑	miR-145	↓	↓	[143]
		ROCK1	↑	miR-129-5p	↓	↓	[144]
		ROCK1	↑	miR-214-5p	↓	↓	[145]
		ROCK1	↑	miR-101	↓	↓	[146]
		ROCK1	↑	miR-202-5p	↓	↓	[147]
Proliferation, migration, invasion		c-Met	↑	miR-449b-5p	↓	↓	[154]
		c-Met	↑	miR-454	↓	↓	[152]
		c-Met	↑	miR-34a	↓	↓	[155]
		c-Met	↑	miR-876-5p	↓	↓	[156]
		AXIN1	↑	miR-31-5p	↓	↓	[158]
Proliferation, migration, apoptosis, EMT	PI3K, Wnt, TGFβ	SOX4	↑	miR-25	↓	↓	[161]
		SOX4	↑	miR-132	↓	↓	[164]
		SOX4	↑	miR-363-3p	↓	↓	[165]
		SOX4	↑	miR-212	↓	↓	[166]
Proliferation		STAT3	↑	miR-340-5p	↓	↓	[167]
		STAT3	↑	miR-30d-5p	↑	↑	[168]
		STAT3	↑	miR-221-3p	↑	↑	[169]
		Dkk-1	↑	miR-107	↓	↓	[171]
		IGF2BP1	↑	miR-150	↓	↓	[172]
		MYC	↑	miR-193b	↓	↓	[174]
Cell cycle	PI3K, Wnt, TGFβ	CCNE2	↑	miR-34c	↓	↓	[178]
	Hippo	PTPRB	↓	miR-624-5p	↑	↓	[184]
	TGFβ	SMAD4	↓	miR-224	↑	↓	[185]
		SMAD7	↑	miR-181c	↓	↓	[191]
		TRAF6	↑	miR-140-3p	↓	↓	[194]
		PTEN and TGF-β1	↓	miR-21	↓	↑	[195]
		TGF-β1		miR-26a-5p		↓	[198]
Migration, invasion		SMAD3	↑	miR-671-5p	↓	↓	[203]
		SMAD3	↑	miR-16-5p	↓	↓	[202]
		TGFβ2	↑	miR-422a	↓	↓	[204]
Metastasis	Notch	NOTCH1, JAG1, HEY2	↑	miR-34c	↓	↓	[219]
		Notch	↑	miR-199b-5p	↓	↓	[221,223]
		Notch2	↑	miR-1296-5p	↓	↓	[224]
	HIF-1/ Rap1/ PI3K-Akt	HIF1α	↑	miR-338-3p	↓	↓	[228]
		HIF1α	↑	miR-186	↓	↓	[229]
Migration, invasion	ErbB	EGFR	↑	miR-7	↓	↓	[234]
		EGFR	↑	miR-3184-5p	↓	↓	[232]
		EGFR (ERK/MAPK)	↑	miR-143	↓	↓	[233]
		EGFR	↑	miR-141-3p	↓	↓	[235]
		EGFR	↑	miR-491-5p	↓	↓	[236]
Metastasis	ErbB	HER2	↑	miR-199b-5p	↑	↑	[222]
	PI3K/Akt/mTOR	PI3KR1	↓	miR-21-5p	↑	↑	[249]
		NRF2	↑	miR-340-5p	↓	↓	[120]
		PI3K, Akt, mTOR	↑	miR-22	↓	↓	[250]
		elF4E	↑	miR-496	↓	↓	[256]
Oncogenesis, angiogenesis, proliferation, metastasis, chemoresistance	PI3K/Akt/mTOR	HMGB1	↑	miR-505	↓	↓	[262,263]
		HMGB1	↑	miR-1284	↓	↓	[264]
		HMGB1	↑	miR-22	↓	↓	[265,266]
		Twist1	↑	miR-22	↓	↓	[267]
		HMGB1	↑	miR-372-3p	↓	↓	[268]
		HMGB1	↑	miR-142-3p	↓	↓	[259]
		HMGB1	↑	miR-129-3p	↓	↓	[259]
		HMGB1	↑	miR-32-5p	↓	↓	[259]
		RUNX2	↑	miR-338-3p	↓	↓	[272]
		CDK4	↑	miR-338-3p	↓	↓	[272]
		MAPK	↑	miR-338-3p	↓	↓	[272]
Chemoresistance		RUNX2	↑	miR-203	↓	↓	[273]
		RUNX2	↑	miR-320b	↓	↓	[274]
		RUNX2	↑	miR-218	↓	↓	[275]
Apoptosis	p53	CDK6, E2F3, Cyclin E2, BCL2	↑	miR-34	↓	↓	[9]
		p53	↓	miR-122-5p	↓	↑	[278]
	PI3K/Akt/mTOR	PI3K/Akt/mTOR	↑	miR-122-5p	↓	↓	[278]
Proliferation, migration, invasion	PI3K/Akt/mTOR	FOXP1	↑	miR-181d-5p	↓	↓	[281]
Apoptosis		Bak	↓	miR-491-5p	↓	↑	[284]
		pro-caspase-9	↑	miR-342-5p	↓	↓	[236,284]
		BCL-2	↓	miR-143	↓	↑	[285]
		Bcl-xL	↑	miR-133a	↓	↓	[290]
	VEGF	VEGF	↑	miR-150-5p	↓	↓	[296]
		VEGF	↑	miR-1	↓	↓	[297]
		VEGF	↑	miR-134	↓	↓	[298]
	PI3K/Akt/mTOR	PTEN	↓	miR-1908	↑	↓	[301]
		PTEN	↓	miR-221	↑	↓	[302]
		PTEN	↓	miR-21	↑	↓	[306]
		PTEN	↓	miR-187	↑	↓	[305]
		PTEN	↓	miR-17	↑	↓	[305]
Proliferation	Hh	Hh	↑	miR-212	↑	↑	[307]
Proliferation, cell cycle, EMT		FOXK1	↑	miR-186-5p	↓	↓	[308]
Proliferation, migration, invasion		NOB1	↑	miR-363	↓	↓	[311]
		TGIF2	↑	miR-541	↓	↓	[314]
		TGIF2	↑	miR-34	↓	↓	[315]
		TGIF2	↑	miR-129	↓	↓	[312]
Metastasis		RECK	↑	miR-21	↓	↓	[317,318]
		RECK	↑	miR-92b	↓	↓	[319]
Antiproliferative effect, proapoptotic		KLF10	↓	miR-197-3p	↑	↓	[321]
Metastasis		HDAC1	↑	miR-326	↓	↓	[322]
		HDAC1	↑	miR-449a	↓	↓	[325]
Proliferation, migration, invasion		HDAC4	↑	miR-637	↓	↓	[328]
Development, metastasis		SND1	↑	miR-296-5p	↓	↓	[332]
Proliferation		FOXP4	↑	miR-491-5p	↓	↓	[334]
Cellular redox homeostasis		GPX4	↑	miR-1287-5p	↓	↓	[336]

## Data Availability

No new data were created or analyzed in this study. Data sharing is not applicable to this article.
